# The impact of primary headaches on disability outcomes: a literature review and meta-analysis to inform future iterations of the Global Burden of Disease study

**DOI:** 10.1186/s10194-024-01735-0

**Published:** 2024-03-04

**Authors:** Marta Waliszewska-Prosół, Danilo Antonio Montisano, Mariola Antolak, Federico Bighiani, Francescantonio Cammarota, Ilaria Cetta, Michele Corrado, Keiko Ihara, Regina Kartamysheva, Igor Petrušić, Maria Magdalena Pocora, Tsubasa Takizawa, Gloria Vaghi, Paolo Martelletti, Barbara Corso, Alberto Raggi, On behalf of the European Headache Federation School of Advanced Studies (EHF-SAS)

**Affiliations:** 1 Department of Neurology, Wroclaw Medical University, Wroclaw, Poland; 2 Dipartimento Di Neuroalgologia, Centro Cefalee, Fondazione IRRCS Istituto Neurologico Carlo Besta, Milan, Italy; 3 Department of Brain and Behavioral Sciences, University of Pavia, Pavia, Italy; 4 Headache Science & Neurorehabilitation Center, IRCCS Mondino Foundation, Pavia, Italy; 5 Neuroimaging Research Unit and Neurology Unit, IRCCS San Raffaele Scientific Institute and Vita-Salute San Raffaele University, Milan, Italy; 6 Department of Neurology, Keio University School of Medicine, Tokyo, Japan; 7 Japanese Red Cross, Ashikaga Hospital, Tochigi, Japan; 8 Department of Neurology, University Clinic of Kazan Federal University, Kazan, Russian Federation; 9 Faculty of Physical Chemistry, Laboratory for Advanced Analysis of Neuroimages, University of Belgrade, Belgrade, Serbia; 10 Unitelma Sapienza University of Rome, Rome, Italy; 11 Neuroscience Institute, National Research Council, Padua, Italy; 12 SC Neurologia, Salute Pubblica, Disabilità, Fondazione IRCCS Istituto Neurologico Carlo Besta, Milan, Italy

**Keywords:** Migraine, Tension-type headache, Cluster headache, Disability, Burden, GBD, YLD

## Abstract

**Background:**

The burden and disability associated with headaches are conceptualized and measured differently at patients’ and populations’ levels. At the patients’ level, through patient-reported outcome measures (PROMs); at population level, through disability weights (DW) and years lived with a disability (YLDs) developed by the Global Burden of Disease Study (GBD). DW are 0–1 coefficients that address health loss and have been defined through lay descriptions. With this literature review, we aimed to provide a comprehensive analysis of disability in headache disorders, and to present a coefficient referring to patients’ disability which might inform future GBD definitions of DW for headache disorders.

**Methods:**

We searched SCOPUS and PubMed for papers published between 2015 and 2023 addressing disability in headache disorders. The selected manuscript included a reference to headache frequency and at least one PROM. A meta-analytic approach was carried out to address relevant differences for the most commonly used PROMs (by headache type, tertiles of medication intake, tertiles of females’ percentage in the sample, and age). We developed a 0–1 coefficient based on the MIDAS, on the HIT-6, and on MIDAS + HIT-6 which was intended to promote future DW iterations by the GBD consortium.

**Results:**

A total of 366 studies, 596 sub-samples, and more than 133,000 single patients were available, mostly referred to cases with migraine. Almost all PROMs showed the ability to differentiate disability severity across conditions and tertiles of medication intake. The indexes we developed can be used to inform future iterations of DW, in particular considering their ability to differentiate across age and tertiles of medication intake.

**Conclusions:**

Our review provides reference values for the most commonly used PROMS and a data-driven coefficient whose main added value is its ability to differentiate across tertiles of age and medication intake which underlie on one side the increased burden due to aging (it is likely connected to the increased impact of common comorbidities), and by the other side the increased burden due to medication consumption, which can be considered as a proxy for headache severity. Both elements should be considered when describing disability of headache disorders at population levels.

**Supplementary Information:**

The online version contains supplementary material available at 10.1186/s10194-024-01735-0.

## Introduction

Headache disorders, according to the Global Burden of Disease Study 2019 (GBD2019), are the third cause of global disability, expressed as years lived with disability (YLDs), in the all range ages and the first in the 15–49 age range [1]. Among the spectrum of “headache disorders'', the GBD includes only migraine and tension-type headache (TTH), whereas medication overuse headache (MOH) burden has been reassigned to migraine and TTH since 2016. The estimated referred to third burdensome primary headache, i.e. cluster headache (CH) gets into the residual category of “other nervous systems diseases”. Notably, migraine represents the second cause overall and the first in young (15-49y) women for YLDs [2–4].

The GBD study represents a comprehensive way to show the prevalence, incidence and burden of headache disorders across countries and age groups, providing sources of information for epidemiological studies that are valid for different regions and age groups. However, it has to be taken into account that it produces estimates based on different sources (i.e. general population censuses, population studies) and not real-world data. Moreover, the meaning of the term “burden” as defined in the GBD architecture is different from that of common sense in the field of headache disorders.

The GBD study reports disease burden with three indicators: years of life lost (YLLs) accounts for premature mortality, YLDs, and disability-adjusted life years (DALYs) a joint measure of YLLs and YLDs which constitute the core measure of burden estimates [1, 2]. Studies on headache disorders, on the contrary, use the term burden to underlie different themes, such as prevalence, impact on work or school activities, interictal burden, and disease costs [5, 6]. Such themes are clearly consistent with the idea of “reduced health state” due to headache and “reduced ability to perform daily life activities”, a kind of concept that is close to the conceptualization of YLDs in the terminology adopted by the GBD. However, there is no mortality associated with headache disorders and the whole burden of headaches is entirely due to non-fatal health outcomes: in other words, YLDs match with DALYs.

YLDs for headache disorders are calculated separately for migraine and TTH as a result of their prevalence, the mean time patients spend with that type of headache multiplied by the associated disability weight (DW). The determination of headache DW [7, 8] was performed through population and internet surveys based on lay descriptions. Migraine lay description was “This person has severe, throbbing head pain, and nausea that cause great difficulty in daily activities and sometimes confine the person to bed. Moving around, light, and noise make it worse.”. TTH lay description was “This person has a moderate headache that also affects the neck, which causes difficulty in daily activities”. Based on such descriptions, DW for migraine was estimated at 0.434 (95% CI: 0.285–0.603), and DW for TTH at 0.036 (95% CI: 0.023–0.053): the interpretation of this is that during an attack the affected person experiences health loss of 43.4% compared with a person in full health in the case of migraine, and of 3.6% in the case of TTH.

Such an approach is quite different from how disability is generally measured in headache research, i.e. through a set of specific questionnaires [9]. The most used disability tool for migraine is the Migraine Disability Assessment (MIDAS) [10], which explores different domains highly tied to patients’ lifestyle, work activity and family role, and strongly related to headache frequency [5]. The second most used tool is the 6-item Headache Impact Test (HIT-6) [11], which also addresses mental functions connected to energy level and emotional functions. Such assessment tools, and the many others used in headache research [8] are useful as outcome measures, but clearly cannot capture the entire experience of living with headache disorders, and other elements should be taken into account for addressing the burden and disability of headache disorders, including the economic implications, work-related costs, including absenteeism, presenteeism and reduced productivity, as well as the interictal burden which is poorly recognized [5, 12–15].

So, the burden of headache disorders is complex to define under one aspect, and the DW definitions are still very partial as drawn on hyper-simplified descriptions. An effort is needed to move from a disease-centered to a patient-centered perspective through the use of Patient Reported Outcome Measures (PROMs) that closely analyze lived experience of people suffering from headache disorders in terms of reduced ability to perform daily activities. In order to pursue this objective, we aimed to present a comprehensive analysis of disability in headache disorders, and to present a coefficient referring to patients’ disability which might inform future GBD definitions of DW specific to headache disorders.

## Methods

We conducted a literature review with meta-analysis and reported results according to the ‘Preferred Reporting Items for Systematic Reviews and Meta-Analyses’ (PRISMA) [16].

### Search strategy

Search terms had to combine information of two main terms, i.e. headache disorders (with possible variations) and disability (with possible variations and the main disability outcomes). PubMed and SCOPUS were searched for the terms, using database-specific variations, in the period between 2015 and 2023.

The PubMed string was: (("migraine"[Title/Abstract] OR "tension type headache"[Title/Abstract] OR "tension-type headache"[Title/Abstract] OR "cluster headache"[Title/Abstract]) AND ("disability"[Title/Abstract] OR "impact"[Title/Abstract] OR "MIDAS"[Title/Abstract] OR "HIT-6"[Title/Abstract] OR "WHODAS"[Title/Abstract] OR "HDI"[Title/Abstract])) AND ((humans[Filter]) AND (2015/1/1:2023/12/31[pdat]) AND (english[Filter])).

The SCOPUS string was: ((TITLE (migraine OR "tension type headache" OR "tension-type headache" OR "cluster headache") AND TITLE (disability OR impact OR midas OR "HIT-6" OR whodas OR hdi))) OR ((ABS (migraine OR "tension type headache" OR "tension-type headache" OR "cluster headache") AND ABS (disability OR impact OR midas OR "HIT-6" OR whodas OR hdi))) AND PUBYEAR > 2014 AND (LIMIT-TO (LANGUAGE, "English")) AND (LIMIT-TO (EXACTKEYWORD, "Human")).

Retrieved references were exported as.csv files and imported to Rayyan QRCI [17] for duplicate checking. The set of records was then exported to MS Excel for study selection and data extraction.

### Study selection

Retrieved references were equally and randomly assigned to the authors who screened titles and abstracts for eligibility. A double check on titles and abstracts eligibility was randomly performed on 20% of selected references: MW-P and AM performed the double check on abstracts.

To be eligible and be evaluated in full texts, records had to be referred to primary research and to report, in titles and abstracts, information on disability associated with one of the three main primary headaches, i.e. migraine, TTH and CH. The diagnoses had to be appointed based on the criteria of the International Headache Society, i.e. the third or third-beta classifications [18, 19], either made by a clinician or through validated screening tools. So we selected records if they: a) mentioned disability evaluation through a PROM; b) were primary research articles. Conversely, we excluded records if they: a) were published before 2015; b) did not have an abstract; c) were not in English; d) were not on primary headaches; e) were letters, editorials, conference material, book chapters, case reports, literature reviews or meta-analyses; f) were clearly out of topic, i.e. clearly not dealing with the topic of disability in primary headaches as referred by patients using some kind of outcome measure. In case of doubts, especially on the last criterion, we decided to keep the record and further re-assess it at the full-text evaluation stage.

In this phase, the agreement among reviewers, i.e. the inter-rater reliability, was calculated using Krippendorff's alpha coefficient (α), which ranges between 0 (total disagreement) and 1 (total agreement). In case of disagreement, the record was considered as selected and retained for full-text evaluation. In case α was below 0.70, a second 20% set of references was submitted to double-check.

Eligible references were equally and randomly assigned to the authors who screened full texts for inclusion. For full texts evaluation, studies were excluded if they: a) could not be retrieved; b) were not in English; c) were not on primary headaches; d) were letters, editorials, conference material, book chapters, case reports, literature reviews or meta-analyses; e) were clearly out of topic, i.e. clearly not dealing with the topic of disability in primary headaches as referred by patients using some kind of outcome measure. In order to be included, studies had to refer information on headache frequency and at least one PROM.

Three authors (MW-P, AM and AR) performed a double check on 20% of the full texts concerning their eligibility and Krippendorff's α was calculated.

### Data extraction

Data extraction was performed through an ad hoc electronic spreadsheet of Microsoft Excel for Windows. Included studies were equally and randomly assigned to the authors who had to extract the following information: a) the number of subjects per main condition, i.e. episodic migraine (EM), chronic migraine (CM), other migraine; episodic TTH, chronic TTH, other TTH; episodic CH, chronic CH, other trigeminal autonomic cephalalgias (TACs); information for the whole number of participants with migraine, TTH, TACs and total subjects were reported; b) the number of females out of the total; c) mean, median, standard deviation, 95% CI or interquartile range for patients’ Age, Headache Frequency, Pain Severity/VAS (visual analog scale 0–10), and Medication Intake; d) mean, median, standard deviation, 95% CI or interquartile range for any possible PROM.

Extracted information was referred to a single time-point, irrespectively of study design (cross-sectional or repeated-measures): in case of cross-sectional studies, the data were extracted as reported in the paper; in case studies with repeated measures (i.e. RCTs or cohort studies), we extracted the information of the baseline evaluations only.

With regard to headache frequency, we intended it on a monthly basis (or 28 days for approximation): if the time frame was different, we marked it and the amount was adjusted using statistical procedures. For studies distinguishing between headache days and migraine days, we recorded headache days. Concerning the medication intake, it was still intended on a monthly/28 day-basis, and considering all drugs together. With regard to PROMs, we moved from a pre-defined list derived from a previous review on the content of PROMs for headache disorders [8], but we added all possible PROMs that were not retained in the list.

The extracted information was at a sub-sample level for each study, as the information on each outcome, i.e. headache frequency, pain severity, and PROMs’ scores, was referred to a specific group of patients. The consequence of this is that, for each included paper, we might have more than one sample: this might be the case of RCTs, in which two or more sub-samples of patients with the same condition were enrolled, or of observational studies in which patients with more than one condition were enrolled. Division in sub-samples was performed each time in which the information on headache frequency and PROMs’ score was available at sub-sample level: if the information was available only at all-patients level, then information on one sample only was extracted.

As a further control measure, we kept full-text selection distinct from data extraction and a “shuffle” procedure was used, i.e. the person who extracted data was not the same who selected the paper. This had the advantage of introducing another control on papers’ eligibility. The choice was made in relation to the large amount of selected papers. In case of disagreement, a senior author (AR) made the final decision.

### Statistical analyses

Studies’ descriptive statistics are reported as mean, standard deviation (SD), median, interquartile range (IQR), and N for quantitative data, and as frequencies and percentages for categorical data.

For the percentage of females and medication intake in each sub-sample, tertiles corresponding variables were created.

#### Meta-analysis

Samples reporting information on the outcome variable, defined as monthly days with headache, were combined using the meta-mean function from the R’s package meta (v 4.0.2) [20]. This function produces a weighted, pooled estimate of the samples’ total mean of monthly days with headache across studies.

In order to take into account possible sources of between-study heterogeneity, as we anticipated due to the remarkable number of samples, a random-effects model was implemented to pool the outcome, and the restricted maximum likelihood estimator [21] was used to calculate the heterogeneity variance τ^2^ (proportion of between-study variance explained by the model). Heterogeneity between the studies was measured through the I2 statistic (with a value higher than 75% considered large).

For studies without information on the outcome’s SD but with available 95%CI, SD was obtained by dividing the length of the confidence interval (upper limit – lower limit) by 3.92, and then multiplying it by the square root of the sample size when this was greater or equal to 100. While, when the sample size was lower than 100, the coefficient 3.92 was replaced by the corresponding value of the t distribution with degrees of freedom equal to the group sample size minus 1.

To identify if a specific heterogeneity pattern is present in the data, subgroup analyses were done considering: headache sub-types (i.e. migraine, TTH and TACs), tertiles of female percentage, age groups (minors vs adults) and tertiles of medication intake.

The same meta-analysis was applied also to the available tools with more than 10 samples (MIDAS, HIT-6, HDI, VAS, WHODAS, WPAI, PedMIDAS, HDI-E, and HDI-P). For these tools, subgroup analyses were implemented when appropriated (i.e. if two or more groups are present).

#### Disability score

In order to inform on disability from different studies in which different outcomes have been used, a coefficient addressing patients’ disability was developed. For selected tools (with adequate sample size) a 0–1 score, where 0 is no disability and 1 is extreme disability, was derived from the predicted values of the regression of the raw tool mean scores over the outcome. To take into account the different size and SD of the included studies, raw mean values were weighted by the tool’s coefficient of variation (test SD/test mean).

One-way ANOVA was applied to inspect the behavior of the scores by subgroup considering: headache sub-types, tertiles of female percentage, age and medication intake.

For this analysis, only the adult samples were included.

## Results

**Fig. 1:**
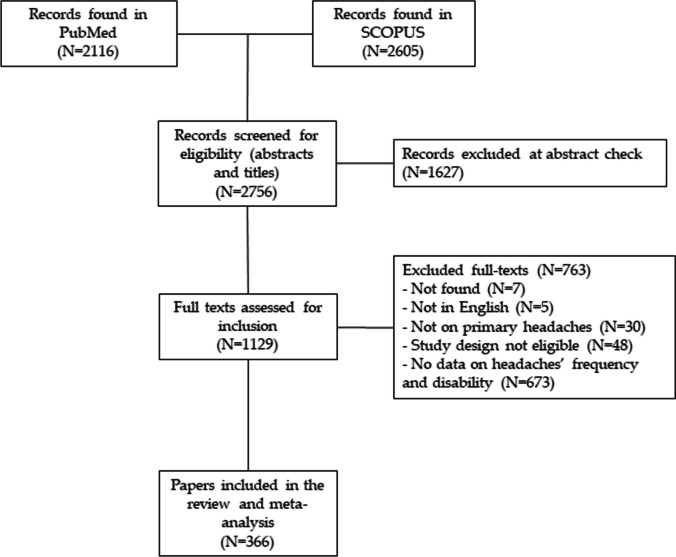
Flowchart of selected studies

Out of 4721 records retrieved in PubMed and SCOPUS, we finally selected 366 papers (see Fig. 1 for PRISMA diagram). The agreement rate at abstract check was 76.5%, at full-texts’ selection was 94.4%. Out of the selected 366 papers, a total of 596 sub-samples (up to seven sub-samples per paper) were identified and used for the analyses (see Supplementary reference list for the full amount of selected papers). Of these, 530 were from the adult’ population (defined as median age ≥ 18y), 28 were from the minors’ population (defined as median age ≤ 18y), whereas in 38 sub-sample age was not specified. Minimum and maximum mean age for minors (26 records) and adults (511 records) were respectively: 8.9 and 15.3, and 18.7 and 70. The remaining 2 records for minors reported only median age with a min of 14 and a max of 15. The same applied to the remaining 19 records for adults, for which min and max median age were 30.8 and 46.

The sub-samples were composed of a minimum of 6 to a maximum of 15,313 subjects, the median number of patients per sample being 86.5. Among minors’ sub-samples, the median was 40 subjects; among adults, it was 87. Patients’ sex was specified in 544 out of 596 sub-samples, and the percentage of females varied from 0 to 100% with a median of 83%: among minors, 62% were females, and among adults 79% were females.

The descriptive number of subjects with different type of Primary Headache is reported in Table 1. In total, data from 133,813 subjects were extracted, of whom 123,098 with migraine, 5,370 with TTH, 1,244 with TACs, and 4,101 with “not better specified” headache disorders.

**Table 1:** Descriptive information on sub-samples features

	**EM**	**CM**	**Other Migraine**	**Migraine Total**	**ETTH**	**CTTH**	**Other TTH**	**TTH Total**	**ECH**	**CCH**	**Other TACs**	**TACs Total**	**Headache Total**	**Headache Frequency**
Min	3	1	5	0	4	1	4	0	12	7	8	0	6	1.7
Max	15,313	1476	567	15,313	495	176	40	1300	47	52	8	659	15,313	87
p25	36	25	31	29.5	10	6	5	0	12	8	8	0	39	6.9
p50	60.5	75	51	74	50	42	22.5	0	29.5	11.5	8	0	86.5	10.9
p75	135	145	111	172	77	74	39.5	0	47	33	8	0	194	19.1
IQR	99	120	80	142.5	67	68	34.5	0	35	25	0	0	155	12.3
Mean	243.6	146.8	103.7	206.5	92.1	52.8	22.2	9.0	29.5	20.5	8	2.1	224.5	13.3
SD	1087.8	223.5	140.5	725.9	142.1	51.4	19.9	66.0	24.7	18.2		29.0	728.8	9.5
N sub-samples	226	209	19	596	17	19	4	596	2	6	1	596	596	536

*EM* Episodic migraine, *CM* Chronic migraine, *ETTH* Episodic TTH, *CTTH* Chronic TTH, *TTH* Tension-type headache, *ECH* Episodic cluster headache, *CCH* Chronic cluster headache, *TACs* Trigeminal autonomic cephalalgias

The Outcome variable, defined as monthly days with headache, ranged from 1.7 to 87 (this value being due to the calculation made for cases with CH, in which single episodes and not days were reported), the median frequency being 10.9, and the mean ± SD of 13.3 ± 9.5. Supplementary Table 1 reports the same information for the subsamples of adults, minors and for those in which age was not specified.

For the composition of the sample by age group, tertile of F%, and tertile of mean medication intake see Supplementary Table 2.

### Meta-analyses

The pooled mean of monthly days with headache estimated by the meta-mean function was 10.6 [10.0 – 11.3] based on 566 samples and 130,280 total subjects. The pooled subgroups’ mean values were: 10.4 for Migraine, 12.1 for Migraine associated with TTH, 41.0 for TACs, and 10.0 for TTH (the value exceeds 30 for TACs as attacks and no days were reported).

We assessed the performance of many PROMs. The majority of the studies used the following evaluation tools: VAS, MIDAS, HIT-6, HDI, WHODAS, WPAI, PedMIDAS, HDI-E, and HDI-P. The NDI, DHI, and Monthly MIDAS were used by 2 studies each, whereas the following tools were employed by only 1 study each: PDI, HEADWORK, AIM-D, PPDI, CHIQ, MIGSEV, and MIBS-4. Almost all PROMs showed the ability to differentiate disability severity across conditions and tertiles of medication intake.

MIDAS data were available from 345 sub-samples and 96,701 subjects, with a pooled mean value of 36.5 [33.8 – 39.5]. The pooled subgroups’ mean values were: 36.8 for Migraine, 154.9 for Migraine associated with TACs, 22.1 for TACs, 31.3 for Migraine associated with TTH and 10.7 for TTH. The high value of MIDAS was reported for the second tertile of Female % and for the high medication intake tertile. For the complete sub-group analysis see the Supplementary Table 3.

HIT-6 data were available from 313 sub-samples and 74,454 subjects, with a pooled mean value of 61.9 [60.6 – 63.1]. The pooled subgroups’ mean values were: 62.3 for Migraine, 69.2 for Migraine associated with TACs, 65.4 for TACs, 65.2 for Migraine associated with TTH and 53.5 for TTH. The highest score was reported for the second tertile of Female % and for the high medication intake sub-groups. For the complete sub-group analysis see Supplementary Table 4.

HDI data were available from 20 sub-samples and 684 subjects, with a pooled mean value of 52.1 [45.3 – 60.4]. The pooled subgroups’ mean values were: 58.1 for Migraine, 48.5 for TTH, 27.2 for Migraine associated with TTH. For the complete sub-group analysis see Supplementary Table 5.

VAS data were available from 289 sub-samples and 42,152 subjects, with a pooled mean value of 7.3 [6.9 – 7.7]. The pooled subgroups’ mean values were: 7.6 for Migraine, 4.7 for TTH, 5.8 for Migraine associated with TTH, 8.1 for TACs and 5.8 for Migraine associated with TACs. The highest score was reported for the third tertile of female % and for the high medication intake sub-group. For the complete sub-group analysis see Supplementary Table 6.

WHODAS data were available from 15 sub-samples and 1,629 subjects, with a pooled mean value of 28.7 [26.0 – 31.6]. The pooled subgroups’ mean values were: 29.6 for Migraine and 17.8 for TACs. The highest score was reported for the second F% tertile. For the complete sub-group analysis see Supplementary Table 7.

WPAI data were available from 11 sub-samples and 4,979 subjects, with a pooled mean value of 16.8 [7.8 – 36.0]. The pooled subgroups’ mean values were: 31.1 for Migraine, 2 for TTH, 4.1 for Migraine associated with TTH. The highest score was reported for the second F% tertile. For the complete sub-group analysis see Supplementary Table 8.

PedMIDAS data were available from 22 sub-samples and 1,520 subjects, with a pooled mean value of 27.9 [21.4 – 36.4]. The pooled subgroups’ mean values were: 32.3 for Migraine, 11.9 for Migraine associated with TTH. The highest score was reported for the second F% tertile. For the complete sub-group analysis see Supplementary Table 9.

HDI is also used with two sub-scores, namely HDI-E (emotional functioning) and HDI-P (physical functioning). HDI-E and HDI-P data were available from 16 sub-samples and 1,678 subjects. HDI-E pooled mean value was 20.0 [18.4 – 21.7]. The pooled subgroups’ mean values were: 27 for Migraine, 19.6 for TTH. HDI-P pooled mean value was 23.9 [22.3 – 25.5]. The pooled subgroups’ mean values were: 34.7 for Migraine, 23.2 for TTH. For the complete sub-group analysis see Supplementary Table 10 and 11.

### Analyses of 0–1 coefficients

In the adults group for each test with sufficient sample size (MIDAS, HIT-6, MIDAS + HIT-6) a 0–1 score was calculated and inspected over different sub-groups (HA type, tertile of female %, tertile of age, and tertiles of medications intake).

Significant differences were found in MIDAS scores between age tertiles (1st vs 3rd, and 2nd vs 3rd), and tertiles of medication intake (1st vs 3rd, 2nd vs 3rd, and 3rd vs Not specified). See supplementary Figs. 1–4.

Significant differences were found in HIT-6 scores between HA subgroups (Migraine vs TACs, and TTH vs TACs), age tertiles (1st vs 2nd, 1st vs 3rd, and 2nd vs 3rd), and tertiles of medication intake (1st vs 2nd, 1st vs 3rd, 2nd vs 3rd, 2nd vs Not specified, and 3rd vs Not specified). See Supplementary Figs. 5–8.

Significant differences were found in MIDAS + HIT-6scores between HA subgroups (Migraine vs TACs, Migraine + TTH vs TACs, TTH vs TACs, and HA type not specified vs TACs), age tertiles (1st vs 2nd, 1st vs 3rd, and 2nd vs 3rd), and tertiles of medication intake (1st vs 2nd, 1st vs 3rd, and 3rd vs Not specified). See Supplementary Figs. 9–12.

For none of the coefficients, any difference was found for the tertiles of females’ percentages.

## Discussion

In this review, we analyzed the response of more than 133,000 patients with primary headaches, derived from 596 single sub-samples and 366 articles, and evaluated the performance of multiple PROMs. The most commonly used scales were the MIDAS, PedMIDAS, HIT-6, VAS, WHODAS, and HDI. Almost all the scales included in our meta-analysis showed the ability to differentiate patient-reported disability levels across primary headaches and tertiles of medication intake, and our results provided reference values that can be used for comparison, in future studies, to address the degree to which enrolled patients show a disability level which can be considered higher or lower that the reference value herein proposed. In addition to this, we presented a data-driven 0–1 coefficient that can be used to inform future iterations of DW for headache disorders, complementing the lay descriptions. The added value of such an index is the ability to differentiate across tertiles of age and medication intake which underlie the increased burden due to age, which is likely due to the increased impact of common comorbidities, and the increased burden due to medication consumption, which can be considered as a proxy for headache severity.

Measurements are an important aspect of scientific research and are intended for use by both physicians and researchers. They can be used to track a patient's progress over time, monitor the effectiveness of treatment and make therapeutic decisions together [22]. PROMs are tools used to assess a patient's health status, symptoms and quality of life. They provide valuable information about the experience of the disease and the patient's perspective [23]. On the other hand, translating an eminently subjective experience like headache into an objective context is extremely difficult and complex. Recent years have shown that there is a great need for universal tools to assess headache-related disability that can be used worldwide. The need to objectify the burden of the disease and the disability associated with it has also been forced upon healthcare systems in the context of social costs associated with reimbursement for expensive therapies, among other things. In many countries, objective improvements in validated questionnaires in primary and secondary endpoints allow for continued treatment [24–26].

Headache disorders can affect various aspects of a patient's life, including social, occupational, recreational and family life. They can cause both increased ictal and interictal burden, as well as economic losses due to lost productivity and medical costs [1, 4, 5]. On the other hand, there seems to be no other such heterogeneous group of diseases as headaches, which, in addition to the natural, individual course associated with periods of exacerbation, are influenced by many external factors [27–29], and a biopsychosocial perspective which integrates external factors to biomedical ones is likely the most adequate to understand such a complexity [30]. Despite the above aspects, more than a dozen tools have been developed to measure the disability, impact and burden of headache and migraine disorders [31, 32].

The most widely used score in clinical practice and research, which is also confirmed by the results of our analysis, in which this scale was used in 345 samples, is MIDAS, which was created to measure and monitor migraine-related disability [22, 33]. The MIDAS is recommended by the IHS for use as an outcome measure for migraine prevention screening in adults. The recall period for MIDAS is 3 months and the questionnaire measures only paroxysmal migraine burden and does not include interictal burden [34]. The score is a combination of responses to five questions, with low scores indicating minimal or no disability and high scores indicating moderate or severe disability. The advantage of this scale is that it takes into account the multiple levels that can be affected by the disease: paid work or school/work, household responsibilities and leisure time. Two additional MIDAS questions address the frequency and severity of headaches [35]. A definite disadvantage for patients who do not keep a headache diary is the need for retrospective analysis of recent months, which in practice is often unreliable and filled out with averaging or "by eye" by many patients. Both in patients with infrequent but very severe attacks as well as frequent but less severe, judgment after time can be erroneous and lead to significant generalizations leading to both overestimation or underestimation of the actual number of days with headaches. In addition, it is problematic to answer the question about work or study in the case of people who are not working for various reasons or are on parental leave. In such a case, the scores are usually underestimated [36].

More than 21 points in MIDAS indicate severe disability, which appears to be an underestimate in the key given the mean value of 36.5 obtained in our analysis of more than 96,000 patients. Similar results much higher than 21 points have been described in large cohort studies that did not report a significant prevalence of patients with chronic migraine [6, 37, 38]. A high MIDAS value was reported for the high medication intake tertile (75.9). This seems obvious because this is the group most likely to suffer from chronic headache complicated by MOH. Many studies confirm the fact of greater disability in patients with higher monthly headache days (MHD), monthly migraine days (MMD) with chronic headaches and abuse of pain medications [39–41].

The PedMIDAS is a pediatric version of the MIDAS and has been validated for ages 4–18. The items are similar to the adult version and the main drawback of this scale is that it is based on the child's memory of the last 3 months, which raises serious questions about the reliability of the score especially in young children [34, 42].

The second most common PROM in our analysis was the Headache Impact Test-6 (HIT-6), which is also recommended by the IHS as one of the secondary endpoints in controlled trials for both episodic and chronic migraine [43]. The questionnaire, consisting of six items, was created to measure the impact of headaches on a person's ability to function in school, work and social situations and covers various aspects of disability from physical pain to emotional suffering [44]. The primary advantage of the HIT-6 is its ease and speed of use and validation in multiple languages but the disadvantages of a recall period of 4 weeks and the large impact of the moment during which it is filled out. For patients who fill it out during a headache attack, scores may be inflated [11, 44]. In our analysis, the highest HIT-6 scores were obtained among patients with TAC-associated migraine (69.2) and for TAC (65.2), and the lowest for TTH (53.5). As with MIDAS, the highest scores were achieved by patients taking large amounts of pain medication. Disability and burden in the course of TAC are among the highest among headache disorders, so it is not surprising that the coexistence of migraine with TAC in the validated scales yields a burden higher than for single diseases [45].

Another very commonly used scale is the visual analog scale (VAS), which has many adherents and thus is very popular and widespread. It has different versions, from those illustrated with appropriate grimaces to those with verbal expressions of pain (verbal scale), which makes it possible to individually choose the appropriate variant for a given patient (depending on age, pain complaints, or character traits, for example) [46]. The main drawback is cited as the difficulty for 10% of patients to understand the extreme values of the section, resulting in an inability to choose the right place in the line, depicting the severity of pain. The scale can also cause difficulties in patients with visual impairment. The VAS scale is not used in children under 5 years of age [47]. In addition, the marking of the score is greatly influenced by the current level of pain and the perception of pain. In our analysis, the highest score on this scale was achieved by TAC patients and the lowest by TTH patients (8.1 vs. 4.7 with a mean of 7.3), confirming previous observations that TACs are among the most severe pains in humans [45].

The WHO Disability Assessment Schedule (WHODAS) is an instrument developed by the World Health Organization to assess functioning, disability and health. With its help, we can comprehensively assess a patient's limitations and difficulties in daily functioning and also evaluate the impact of health interventions on the level of disability understood holistically, in the context of the patient's living environment before and after the intervention [48].

In our analysis, this scale was frequently used to assess disability in migraine patients and the highest score was recorded for the second tertiles of women percentage in the samples. The WHODAS has the advantages of high reliability and accuracy, but it also has some limitations. Some questions may be difficult to understand or answer for people with low levels of education or culturally and racially inappropriate. It is also a generic tool that does not take into account the specifics of different diseases or disorders and the factors that influence them [49].

### PROMS limitations

Validated questionnaires are crucial for assessing headache outcomes, but they show significant limitations in real-world clinical practice. Monitoring patients with PROMS is less reliable than expected and selecting the ideal set of short and non-intrusive tests to provide useful information is very difficult. Gil-Gouveia et al. showed that patients in a PROM assessment can give different answers to the same question asked minutes apart [50]. This is most likely related to various factors such as interpretive difficulties, educational status, long-term motivation and secondary benefits (e.g., access to reimbursable treatment) but also concentration abilities and current pain status [51].

On the other hand, it is very difficult to define clinically a change in PROMs scores because the more than 50% improvement expected and required by the IHS very often does not translate into real improvement and does not significantly reduce disability [34, 52]. Often, improving on one aspect and achieving one goal does not mean solving the whole problem and the patient's priority may change and direct him towards another goal. Then it would be reasonable to use other scales that assess other individual variables [53]. Therefore, another challenge in developing a reliable tool for assessing the course of headache-related disorders is to identify a minimal important change (MIC) value that would be a reliable indicator of a patient's real clinical improvement. MIC is "*the smallest change in a treatment outcome that an individual patient would identify as important and which would indicate a change in the patient's management*" and so this is another multidimensional and difficult concept. [34, 53, 54].

Analyzing the performance of multiple PROMs, we developed a coefficient on patient disability to obtain information from different studies and then analyzed it in different subgroups. We showed that its value for the MIDAS, HIT-6 and MIDAS + HIT-6 scales was higher in patients suffering from two types of headaches, those taking pain medications more often, and the elderly. We believe that the reference value of a 0–1 coefficient could be used in a future iteration of these GBD for the disability weights for headache disorders. Its purpose would be to determine the disability weights for headaches, which are currently based on an assessment that a given amount of persons gave to lay descriptions of migraine and TTH status.

The herein-developed coefficients are not intended to replace DW, but to inform on possible other ways to produce it based on data derived from patients. The three coefficients showed an association with age tertiles, which is likely mediated by the presence of comorbidities. Comorbidities play a central role in populations’ disability [55, 56] and headache sufferers are prone to experience a wide set of common comorbidities (including low back pain, chronic pelvic pain, fibromyalgia, anxiety, depression, and diabetes) [57], which have been shown to explain 65% of disability associated with migraine [58]. Each of these conditions is main driver of disability, as shown by GBD 2019 estimates [2]: low back pain ranks first (7.4% of all-cause YLDs), depressive disorders rank second (5.4%), diabetes ranks sixth (4.2%), and anxiety disorders rank eighth (3.3%). The GBD does not take into account the joint effect of different diseases, but it is clear that, at a single patients’ level, the presence of comorbidities determines an additional impact. The situation of multimorbidity (i.e. the joint presence of two/three or more chronic conditions, depending on different definitions [59]) is a major driver of reduced health, which has been shown to impact disability in patients with chronic migraine associated with medication overuse [60]. The consumption of medications for acute headache treatment is the second added value of our coefficients: future iteration of DW should take into account the amount of consumed drugs, as it is both a proxy for increased headache severity and for the risk of developing medication overuse headache (MOH), which constitute a relevant health concern. Treating MOH is a core business of headache care, and the importance of detoxification towards improvement in patients’ health and disability has been widely proven [61–63].

Some limitations have to be taken into consideration. First, we were unable to locate seven studies. Second, there was a considerable heterogeneity in meta-analysis results: this however can be interpreted as just a matter of fact, in consideration of the wide differences between samples in terms of size, conditions (although the vast majority was on migraine) and employed outcomes. Third, we did not have track of the presence and type of prophylaxis, and the kind of drugs used for acute consumption. A more focused set of selection criteria would have probably produced more homogeneous results, but at the price of looking at the variety of clinical situations that was herein included.

## Conclusions

In summary, headaches are associated with significant disability affecting many aspects of patients' lives. Objectification of this impact is crucial for monitoring treatment effects and making management decisions. Our meta-analysis showed that there are several scales readily used in clinical trials and clinical practice. It is important to remember that none of these tools fully captures the entire experience of headache disability. Each score has its advantages but also its disadvantages, and should only be taken as a certain benchmark that slightly exceeds the number of days with headaches. It should also be emphasized that in the case of headaches, the term "burden" refers to many aspects of the disease, which additionally has its own specific, irregular course dependent on many factors. As a result, reliable "measurability" of the disability is very difficult, all the more so because the questionnaires to date do not refer to inter-ictal periods during which patients also suffer negative effects of the disease. There is a need for further development and operationalization of scales and coefficients that, both population-wise and individually, will allow the objectification of headache-related disability.

### Supplementary Information

**Supplementary Material 1.**

**Supplementary Material 2.**

## Data Availability

Not applicable.

## Abbreviations

AIM-D: The Activity Impairment in Migraine Diary
CH: Cluster headache
CHIQ: Cluster Headache Impact Questionnaire
DALYs: Disability-adjusted life years
DW: Disability weight
GBD: Global Burden of Disease
HDI: Headache-Disability Inventory
HIT-6: Headache Impact Test 6
HIS: International Headache Society
IQR: Interquartile range
MIBS-4: Migraine Interictal Burden Scale
MIC: Minimal important change
MIDAS: Migraine Disability Assessment
MOH: Medication overuse headache
NDI: Neck Disability Index
PDI: Pain Disability Index
PPDI: Pediatric pain disability score
PRISMA: Preferred Reporting Items for Systematic Reviews and Meta-Analyses
PROMs: Patient Reported Outcome Measures
SD: Standard deviation
TAC: Trigeminal autonomic cephalalgias
TTH: Tension-type headache
VAS: Visual analogue scale
WHODAS: The WHO Disability Assessment Schedule
WPAI: Work Productivity and Activity Impairment
YLDs: Years lived with disability
