# Supplementary material for: The impact of primary headaches on disability outcomes: a literature review and meta-analysis to inform future iterations of the Global Burden of Disease study
Source: J Headache Pain. 2024 Mar 4;25(1):27. doi: 10.1186/s10194-024-01735-0 (PMC10910736; doi:10.1186/s10194-024-01735-0)
Supplement: Supplementary file 1 — Supplementary Material 1. [file 10194_2024_1735_MOESM1_ESM.docx]

Supplementary Table 1. Descriptive analyses for subsamples of minors, adults and of non-specified age subjects.

|  | **EM** | **CM** | **Other M** | **Migraine TOT** | **ETTH** | **CTTH** | **Other TTH** | **TTHTOT** | **ECH** | **CCH** | **Other TACs** | **TACs TOT** | **Headache TOT** | **Headache Frequency** |
| --- | --- | --- | --- | --- | --- | --- | --- | --- | --- | --- | --- | --- | --- | --- |
| **Minors** | | | | | | | | | | | | | | |
| Min | 12 | 15 | . | 0 | . | . | . | 0 | . | . | . | 0 | 9 | 2.3 |
| Max | 92 | 47 | . | 361 | . | . | . | 47 | . | . | . | 0 | 361 | 21.1 |
| p25 | 29 | 15 | . | 27.5 | . | . | . | 0 | . | . | . | 0 | 28.5 | 9.3 |
| p50 | 30 | 31 | . | 38.5 | . | . | . | 0 | . | . | . | 0 | 40 | 12.3 |
| p75 | 40 | 47 | . | 49 | . | . | . | 0 | . | . | . | 0 | 63 | 15.9 |
| IQR | 11 | 32 | . | 21.5 | . | . | . | 0 | . | . | . | 0 | 34.5 | 6.6 |
| Mean | 37.5 | 31.0 | . | 64.0 | . | . | . | 2.4 | . | . | . | 0 | 68.6 | 12.5 |
| SD | 18.9 | 22.6 | . | 81.9 | . | . | . | 9.1 | . | . | . | 0 | 85.1 | 4.6 |
| N sub-samples | 13 | 2 | 0 | 28 | 0 | 0 | 0 | 28 | 0 | 0 | 0 | 28 | 28 | 24 |
| **Adults** | | | | | | | | | | | | | | |
| Min | 3 | 1 | 5 | 0 | 4 | 1 | 4 | 0 | 12 | 7 | 8 | 0 | 7 | 1.7 |
| Max | 15313 | 1476 | 567 | 15313 | 495 | 176 | 40 | 1300 | 47 | 33 | 8 | 196 | 15313 | 87 |
| p25 | 36 | 25.5 | 31 | 30 | 10 | 6 | 5 | 0 | 12 | 8 | 8 | 0 | 40 | 6.9 |
| p50 | 64.5 | 73.5 | 51 | 76 | 50 | 42 | 22.5 | 0 | 29.5 | 10 | 8 | 0 | 87 | 10.7 |
| p75 | 168 | 149.5 | 111 | 187 | 77 | 74 | 39.5 | 0 | 47 | 13 | 8 | 0 | 199 | 19.4 |
| IQR | 132 | 124 | 80 | 157 | 67 | 68 | 34.5 | 0 | 35 | 5 | 0 | 0 | 159 | 12.5 |
| Mean | 260.9 | 149.7 | 103.7 | 218.6 | 92.1 | 52.8 | 22.2 | 10.0 | 29.5 | 14.2 | 8 | 0.7 | 236.9 | 13.4 |
| SD | 1148.0 | 227.55 | 140.5 | 766.2 | 142.1 | 51.4 | 19.9 | 69.9 | 24.7 | 10.8 | . | 9.4 | 768.9 | 9.0 |
| N sub-samples | 202 | 200 | 19 | 530 | 17 | 19 | 4 | 530 | 2 | 5 | 1 | 530 | 530 | 481 |
| **Age not specified** | | | | | | | | | | | | | | |
| Min | 43 | 6 | . | 0 | . | . | . | 0 | . | 52 | . | 0 | 6 | 1.8 |
| Max | 936 | 279 | . | 1175 | . | . | . | 0 | . | 52 | . | 659 | 1175 | 85 |
| p25 | 48 | 14 | . | 38 | . | . | . | 0 | . | 52 | . | 0 | 43 | 2.7 |
| p50 | 97 | 95 | . | 85 | . | . | . | 0 | . | 52 | . | 0 | 96 | 8.6 |
| p75 | 137 | 107 | . | 143 | . | . | . | 0 | . | 52 | . | 0 | 160 | 15.2 |
| IQR | 89 | 93 | . | 105 | . | . | . | 0 | . | 0 | . | 0 | 117 | 12.5 |
| Mean | 168.8 | 98.7 | . | 143.4 | . | . | . | 0 | . | 52 | . | 22.4 | 165.7 | 13.4 |
| SD | 257.2 | 89.8 | . | 230.4 | . | . | . | 0 | . | . | . | 108.7 | 241.5 | 17.0 |
| N sub-samples | 11 | 7 | 0 | 38 | 0 | 0 | 0 | 38 | 0 | 1 | 0 | 38 | 38 | 31 |

Table 2: Composition of the sample by age group, Tertile of F%, tertile of mean medication intake

| \| Age group \| minors \| adults \| NA \| Total \| \| --- \| --- \| --- \| --- \| --- \| \|  \| (N = 28) \| (N = 530) \| (N = 38) \| (N = 596) \| |
| --- | --- | --- | --- | --- | --- | --- | --- | --- | --- | --- |
| \| **HA type macro** \|  \|  \|  \|  \| \| --- \| --- \| --- \| --- \| --- \| \| no HA type data info \| 1 (3.6%) \| 6 (1.1%) \| 0 (0.0%) \| 7 (1.2%) \| \| Migraine \| 23 (82.1%) \| 468 (88.3%) \| 35 (92.1%) \| 526 (88.3%) \| \| TTH \| 0 (0.0%) \| 38 (7.2%) \| 0 (0.0%) \| 38 (6.4%) \| \| TACs \| 0 (0.0%) \| 6 (1.1%) \| 3 (7.9%) \| 9 (1.5%) \| \| Migraine+TTH \| 4 (14.3%) \| 11 (2.1%) \| 0 (0.0%) \| 15 (2.5%) \| \| Migraine+TACs \| 0 (0.0%) \| 1 (0.2%) \| 0 (0.0%) \| 1 (0.2%) \| |
| \| F% tertile \| NA \| 1 tertile \| 2 tertile \| 3 tertile \| Total \| \| --- \| --- \| --- \| --- \| --- \| --- \| \|  \| (N = 52) \| (N = 182) \| (N = 181) \| (N = 181) \| (N = 596) \| |
| \| **HA type macro** \|  \|  \|  \|  \|  \| \| --- \| --- \| --- \| --- \| --- \| --- \| \| no HA type data info \| 2 (3.8%) \| 2 (1.1%) \| 1 (0.6%) \| 2 (1.1%) \| 7 (1.2%) \| \| Migraine \| 47 (90.4%) \| 133 (73.1%) \| 177 (97.8%) \| 169 (93.4%) \| 526 (88.3%) \| \| TTH \| 2 (3.8%) \| 30 (16.5%) \| 1 (0.6%) \| 5 (2.8%) \| 38 (6.4%) \| \| TACs \| 1 (1.9%) \| 7 (3.8%) \| 0 (0.0%) \| 1 (0.6%) \| 9 (1.5%) \| \| Migraine+TTH \| 0 (0.0%) \| 9 (4.9%) \| 2 (1.1%) \| 4 (2.2%) \| 15 (2.5%) \| \| Migraine+TACs \| 0 (0.0%) \| 1 (0.5%) \| 0 (0.0%) \| 0 (0.0%) \| 1 (0.2%) \| |
| \| Medication use tertile \| 1 \| 2 \| 3 \| Total \| \| --- \| --- \| --- \| --- \| --- \| \|  \| (N = 35) \| (N = 35) \| (N = 35) \| (N = 105) \| |
| \| **HA type macro** \|  \|  \|  \|  \| \| --- \| --- \| --- \| --- \| --- \| \| no HA type data info \| 0 (0.0%) \| 0 (0.0%) \| 0 (0.0%) \| 7 (1.2%) \| \| Migraine \| 34 (97.1%) \| 33 (94.3%) \| 34 (97.1%) \| 526 (88.3%) \| \| TTH \| 1 (2.9%) \| 0 (0.0%) \| 0 (0.0%) \| 38 (6.4%) \| \| TACs \| 0 (0.0%) \| 1 (2.9%) \| 1 (2.9%) \| 9 (1.5%) \| \| Migraine+TTH \| 0 (0.0%) \| 0 (0.0%) \| 0 (0.0%) \| 15 (2.5%) \| \| Migraine+TACs \| 0 (0.0%) \| 1 (2.9%) \| 0 (0.0%) \| 1 (0.2%) \| |

Table 3. Meta-analysis for MIDAS data.

| Sub-group | k | mean |  | 95%-CI | tau^2 | tau | Q | I^2 | P^a^_sub_ | P^b^_sub_ |
| --- | --- | --- | --- | --- | --- | --- | --- | --- | --- | --- |
| HA type |  |  |  |  |  |  |  |  | <0.0001 | <0.0001 |
| Migraine | 306 | 36.7856 | [ 33.9464; | 39.8622] | 0.5041 | 0.71 | 159297.8 | 99.80% |  |  |
| Migraine+TTH | 5 | 31.2943 | [ 13.8336; | 70.7939] | 0.8636 | 0.9293 | 1294.58 | 99.70% |  |  |
| TACs | 3 | 22.0983 | [ 15.5769; | 31.3499] | 0.0911 | 0.3019 | 84.89 | 97.60% |  |  |
| TTH | 2 | 10.7268 | [ 10.0556; | 11.4427] | 0 | 0 | 0.71 | 0.00% |  |  |
| Migraine+TACs | 1 | 154.91 | [133.8616; | 179.2680] | -- | -- | 0 | -- |  |  |
| no HA type data | 5 | 40.4442 | [ 27.2298; | 60.0714] | 0.2005 | 0.4478 | 206.36 | 98.10% |  |  |
| Tertiles of F% |  |  |  |  |  |  |  |  | 0.0062 | 0.0025 |
| Tertile 1 | 80 | 29.8408 | [25.0838; | 35.5000] | 0.613 | 0.783 | 32944.59 | 99.80% |  |  |
| Tertile 2 | 115 | 42.5423 | [37.8168; | 47.8583] | 0.4087 | 0.6393 | 108301.9 | 99.90% |  |  |
| Tertile 3 | 94 | 34.8031 | [30.2339; | 40.0628] | 0.4736 | 0.6882 | 17436.52 | 99.50% |  |  |
| Tertile NA | 33 | 40.0796 | [31.0190; | 51.7868] | 0.5536 | 0.7441 | 3395.18 | 99.10% |  |  |
| Age |  |  |  |  |  |  |  |  | 0.1848 | 0.0665 |
| minors | 4 | 21.7654 | [12.5118; | 37.8629] | 0.2734 | 0.5229 | 113.2 | 97.30% |  |  |
| adults | 294 | 36.7464 | [33.9049; | 39.8259] | 0.4859 | 0.6971 | 156815.1 | 99.80% |  |  |
| NA | 24 | 37.0715 | [25.7380; | 53.3955] | 0.8235 | 0.9074 | 6068.58 | 99.60% |  |  |
| Tertiles of Medication intake |  |  |  |  |  |  |  |  | <0.0001 | <0.0001 |
| Tertile 1 | 24 | 27.6635 | [23.2417; | 32.9266] | 0.1824 | 0.4271 | 4963.68 | 99.50% |  |  |
| Tertile 2 | 20 | 51.4805 | [38.6151; | 68.6324] | 0.4212 | 0.649 | 1535.11 | 98.80% |  |  |
| Tertile 3 | 23 | 75.9316 | [61.1420; | 94.2987] | 0.2715 | 0.5211 | 585.86 | 96.20% |  |  |
| Tertile NA | 255 | 34.1953 | [31.3246; | 37.3290] | 0.4998 | 0.707 | 133382.8 | 99.80% |  |  |

Note. k: number of sub-samples; mean: pooled mean; P^a^_sub_: p-value of the difference in mean values between all the sub-groups; P^b^_sub_: p-value of the difference in mean values between the sub-groups, without taking into account “NA” category

Table 4. Meta-analysis for HIT-6 data.

| Sub-group | k | mean |  | 95%-CI | tau^2 | tau | Q | I^2 | P^a^_sub_ | P^b^_sub_ |
| --- | --- | --- | --- | --- | --- | --- | --- | --- | --- | --- |
| HA type |  |  |  |  |  |  |  |  | <0.0001 | <0.0001 |
| Migraine | 270 | 62.3021 | [61.0037; | 63.6281] | 0.0309 | 0.1758 | 82000.63 | 99.70% |  |  |
| Migraine+TTH | 2 | 65.2368 | [62.6547; | 67.9253] | 0.0005 | 0.0216 | 2.21 | 54.70% |  |  |
| TACs | 7 | 65.3968 | [63.5528; | 67.2944] | 0.0012 | 0.0351 | 76.91 | 92.20% |  |  |
| TTH | 18 | 53.5525 | [50.5389; | 56.7458] | 0.015 | 0.1227 | 1473.1 | 98.80% |  |  |
| Migraine+TACs | 1 | 69.17 | [67.3423; | 71.0473] | -- | -- | 0 | -- |  |  |
| no HA type data | 1 | 65.9 | [64.9485; | 66.8654] | -- | -- | 0 | -- |  |  |
| Tertiles of F% |  |  |  |  |  |  |  |  | 0.0002 | <0.0001 |
| Tertile 1 | 95 | 58.8 | [56.7101; | 60.9670] | 0.032 | 0.1789 | 16901.89 | 99.40% |  |  |
| Tertile 2 | 87 | 64.0581 | [63.2414; | 64.8854] | 0.0035 | 0.0595 | 19579.99 | 99.60% |  |  |
| Tertile 3 | 94 | 62.5659 | [59.6250; | 65.6519] | 0.0565 | 0.2377 | 46569.37 | 99.80% |  |  |
| Tertile NA | 23 | 63.7497 | [62.5287; | 64.9945] | 0.002 | 0.0452 | 208.78 | 89.50% |  |  |
| Age |  |  |  |  |  |  |  |  | 0.7167 | 0.9414 |
| Minors | 2 | 61.5974 | [56.3794; | 67.2983] | 0.0032 | 0.0564 | 4.14 | 75.90% |  |  |
| Adults | 279 | 61.8081 | [60.5172; | 63.1265] | 0.0321 | 0.179 | 90274.27 | 99.70% |  |  |
| NA | 18 | 62.7956 | [60.7719; | 64.8867] | 0.0049 | 0.0701 | 819.69 | 97.90% |  |  |
| Tertiles of Medication intake |  |  |  |  |  |  |  |  | <0.0001 | <0.0001 |
| Tertile 1 | 18 | 61.8617 | [60.3476; | 63.4138] | 0.0027 | 0.0517 | 839.75 | 98.00% |  |  |
| Tertile 2 | 21 | 65.0119 | [63.4805; | 66.5802] | 0.003 | 0.0544 | 1613.78 | 98.80% |  |  |
| Tertile 3 | 23 | 65.9038 | [65.1297; | 66.6872] | 0.0007 | 0.0266 | 324 | 93.20% |  |  |
| Tertile NA | 237 | 61.2119 | [59.7254; | 62.7353] | 0.037 | 0.1923 | 75292.65 | 99.70% |  |  |

Note. k: number of sub-samples; mean: pooled mean; P^a^_sub_: p-value of the difference in mean values between all the sub-groups; P^b^_sub_: p-value of the difference in mean values between the sub-groups, without taking into account “NA” category

Table 5. Meta-analysis for HDI data.

| Sub-group | k | Mean |  | 95%-CI | tau^2 | tau | Q | I^2 | P^a^_sub_ |
| --- | --- | --- | --- | --- | --- | --- | --- | --- | --- |
| HA type |  |  |  |  |  |  |  |  | <0.0001 |
| Migraine | 14 | 58.1074 | [52.0055; | 64.9254] | 0.0424 | 0.206 | 231.41 | 94.40% |  |
| Migraine+TTH | 2 | 27.161 | [25.0257; | 29.4786] | 0 | 0 | 0.59 | 0.00% |  |
| TACs | 0 | NA |  |  | -- | -- | -- | -- |  |
| TTH | 2 | 48.46 | [41.2364; | 56.9488] | 0 | 0 | 0.01 | 0.00% |  |
| Migraine+TACs | 0 | NA |  |  | -- | -- | -- | -- |  |
| no HA type data | 0 | NA |  |  | -- | -- | -- | -- |  |
| Tertiles of F% |  |  |  |  |  |  |  |  | 0.9095 |
| Tertile 1 | 4 | 48.8487 | [31.5378; | 75.6614] | 0.1935 | 0.4398 | 234.5 | 98.70% |  |
| Tertile 2 | 6 | 54.0576 | [46.4276; | 62.9416] | 0.0332 | 0.1821 | 110.44 | 95.50% |  |
| Tertile 3 | 8 | 52.8841 | [41.5319; | 67.3393] | 0.1181 | 0.3437 | 296.23 | 97.60% |  |
| Tertile NA | 0 | NA |  |  | -- | -- | -- | -- |  |
| Age |  |  |  |  |  |  |  |  | 0.0065 |
| Minors | 0 | NA |  |  | -- | -- | -- | -- |  |
| Adults | 16 | 50.8342 | [43.4627; | 59.4559] | 0.0984 | 0.3137 | 650 | 97.70% |  |
| NA | 2 | 65.4127 | [59.6675; | 71.7110] | 0.0005 | 0.0222 | 1.12 | 11.10% |  |
| Tertiles of Medication intake |  |  |  |  |  |  |  |  | -- |
| Tertile 1 | 0 | NA |  |  | -- | -- | -- | -- |  |
| Tertile 2 | 0 | NA |  |  | -- | -- | -- | -- |  |
| Tertile 3 | 0 | NA |  |  | -- | -- | -- | -- |  |
| Tertile NA | 18 | 52.3129 | [45.2962; | 60.4165] | 0.0934 | 0.3055 | 651.83 | 97.4% |  |

Note. k: number of sub-samples; mean: pooled mean; P^a^_sub_: p-value of the difference in mean values between all the sub-groups;

Table 6. Meta-analysis for VAS data.

| Sub-group | k | Mean |  | 95%-CI | tau^2 | tau | Q | I^2 | P^a^_sub_ | P^b^_sub_ |
| --- | --- | --- | --- | --- | --- | --- | --- | --- | --- | --- |
| HA type |  |  |  |  |  |  |  |  | <0.0001 | <0.0001 |
| Migraine | 223 | 7.616 | [7.2092; | 8.0457] | 0.1735 | 0.4165 | 63120.88 | 99.60% |  |  |
| Migraine+TTH | 6 | 5.8553 | [4.8015; | 7.1404] | 0.0557 | 0.2359 | 128.41 | 96.10% |  |  |
| TACs | 5 | 8.1412 | [7.6203; | 8.6977] | 0.0049 | 0.0697 | 67.68 | 94.10% |  |  |
| TTH | 16 | 4.7157 | [3.8982; | 5.7045] | 0.1455 | 0.3814 | 1792.2 | 99.20% |  |  |
| Migraine+TACs | 0 | NA |  |  | -- | -- | -- | -- |  |  |
| no HA type data | 2 | 6.1976 | [5.8178; | 6.6023] | 0.002 | 0.0449 | 30.92 | 96.80% |  |  |
| Tertiles of F% |  |  |  |  |  |  |  |  | 0.0949 | 0.0558 |
| Tertile 1 | 97 | 6.8053 | [6.2444; | 7.4165] | 0.1847 | 0.4298 | 17788.45 | 99.50% |  |  |
| Tertile 2 | 61 | 7.521 | [6.9102; | 8.1859] | 0.1129 | 0.336 | 16697.82 | 99.60% |  |  |
| Tertile 3 | 73 | 8.0607 | [7.1739; | 9.0571] | 0.2565 | 0.5064 | 32166.17 | 99.80% |  |  |
| Tertile NA | 21 | 7.1142 | [6.7078; | 7.5452] | 0.0163 | 0.1275 | 227.74 | 91.20% |  |  |
| Age |  |  |  |  |  |  |  |  | 0.9524 | 0.7640 |
| Minors | 10 | 7.2048 | [6.3404; | 8.1870] | 0.0397 | 0.1992 | 359.55 | 97.50% |  |  |
| Adults | 225 | 7.3614 | [6.9457; | 7.8020] | 0.1963 | 0.443 | 66476.38 | 99.70% |  |  |
| NA | 17 | 7.3086 | [6.8813; | 7.7625] | 0.0139 | 0.1181 | 446.97 | 96.40% |  |  |
| Tertiles of Medication intake |  |  |  |  |  |  |  |  | 0.0054 | 0.0057 |
| Tertile 1 | 20 | 6.9086 | [6.3611; | 7.5032] | 0.035 | 0.187 | 3880.17 | 99.50% |  |  |
| Tertile 2 | 9 | 7.3211 | [6.4566; | 8.3012] | 0.0351 | 0.1875 | 253.04 | 96.80% |  |  |
| Tertile 3 | 13 | 7.9524 | [7.6809; | 8.2335] | 0.0035 | 0.0591 | 192.76 | 93.80% |  |  |
| Tertile NA | 210 | 7.3495 | [6.9072; | 7.8201] | 0.2087 | 0.4568 | 62792.37 | 99.70% |  |  |

Note. k: number of sub-samples; mean: pooled mean; P^a^_sub_: p-value of the difference in mean values between all the sub-groups; P^b^_sub_: p-value of the difference in mean values between the sub-groups, without taking into account “NA” category

Table 7. Meta-analysis for WHODAS data.

| Sub-group | k | mean |  | 95%-CI | tau^2 | tau | Q | I^2 | P^a^_sub_ |
| --- | --- | --- | --- | --- | --- | --- | --- | --- | --- |
| HA type |  |  |  |  |  |  |  |  | <0.0001 |
| Migraine | 14 | 29.6 | [27.2225; | 32.1852] | 0.0228 | 0.1509 | 146.68 | 91.10% |  |
| Migraine+TTH | 0 | NA |  |  | -- | -- | -- | -- |  |
| TACs | 1 | 17.8 | [14.5257; | 21.8123] | -- | -- | 0 | -- |  |
| TTH | 0 | NA |  |  | -- | -- | -- | -- |  |
| Migraine+TACs | 0 | NA |  |  | -- | -- | -- | -- |  |
| no HA type data | 0 | NA |  |  | -- | -- | -- | -- |  |
| Tertiles of F% |  |  |  |  |  |  |  |  | <0.0001 |
| Tertile 1 | 1 | 17.8 | [14.5257; | 21.8123] | -- | -- | 0 | -- |  |
| Tertile 2 | 11 | 31.064 | [28.8553; | 33.4417] | 0.014 | 0.1183 | 105.66 | 90.50% |  |
| Tertile 3 | 3 | 23.5393 | [20.6557; | 26.8255] | 0.0067 | 0.0818 | 4.03 | 50.30% |  |
| Tertile NA | 0 | NA |  |  | -- | -- | -- | -- |  |
| Age |  |  |  |  |  |  |  |  | -- |
| Minors | 0 | NA |  |  | -- | -- | -- | -- |  |
| Adults | 15 | 28.7219 | [26.0482; | 31.6701] | 0.0339 | 0.1842 | 172.87 | 91.90% |  |
| NA | 0 | NA |  |  | -- | -- | -- | -- |  |
| Tertiles of Medication intake |  |  |  |  |  |  |  |  | 0.0792 |
| Tertile 1 | 0 | NA |  |  | -- | -- | -- | -- |  |
| Tertile 2 | 0 | NA |  |  | -- | -- | -- | -- |  |
| Tertile 3 | 1 | 25.3 | [22.6494; | 28.2608] | -- | -- | 0 | -- |  |
| Tertile NA | 14 | 28.973 | [26.1314; | 32.1237] | 0.0354 | 0.1882 | 162.79 | 92.00% |  |

Note. k: number of sub-samples; mean: pooled mean; P^a^_sub_: p-value of the difference in mean values between all the sub-groups;

Table 8. Meta-analysis for WPAI data.

| Sub-group | k | mean |  | 95%-CI | tau^2 | tau | Q | I^2 | P^a^_sub_ | P^b^_sub_ |
| --- | --- | --- | --- | --- | --- | --- | --- | --- | --- | --- |
| HA type |  |  |  |  |  |  |  |  | <0.0001 | <0.0001 |
| Migraine | 7 | 31.1301 | [16.6404; | 58.2367] | 0.7134 | 0.8447 | 4415.26 | 99.90% |  |  |
| Migraine+TTH | 1 | 4.1 | [ 3.5548; | 4.7288] | -- | -- | 0 | -- |  |  |
| TACs | 0 | NA |  |  | -- | -- | -- | -- |  |  |
| TTH | 1 | 2 | [ 1.8890; | 2.1175] | -- | -- | 0 | -- |  |  |
| Migraine+TACs | 0 | NA |  |  | -- | -- | -- | -- |  |  |
| no HA type data | 2 | 11.5048 | [ 1.1172; | 118.4716] | 2.8301 | 1.6823 | 2837.26 | 100.00% |  |  |
| Tertiles of F% |  |  |  |  |  |  |  |  | 0.0002 | -- |
| Tertile 1 | 5 | 5.4919 | [ 2.0655; | 14.6024] | 1.2428 | 1.1148 | 4838.18 | 99.90% |  |  |
| Tertile 2 | 3 | 45.7818 | [36.4474; | 57.5067] | 0.0384 | 0.196 | 52.79 | 96.20% |  |  |
| Tertile 3 | 3 | 40.1753 | [37.7277; | 42.7817] | 0.0025 | 0.0503 | 11.43 | 82.50% |  |  |
| Tertile NA | 0 | NA |  |  | -- | -- | -- | -- |  |  |
| Age |  |  |  |  |  |  |  |  | -- | -- |
| Minors | 0 | NA |  |  | -- | -- | -- | -- |  |  |
| Adults | 11 | 16.8381 | [7.8664; | 36.0422] | 1.6569 | 1.2872 | 15656.89 | 99.90% |  |  |
| NA | 0 | NA |  |  | -- | -- | -- | -- |  |  |
| Tertiles of Medication intake |  |  |  |  |  |  |  |  | 0.0171 | -- |
| Tertile 1 | 0 | NA |  |  | -- | -- | -- | -- |  |  |
| Tertile 2 | 0 | NA |  |  | -- | -- | -- | -- |  |  |
| Tertile 3 | 2 | 40.7977 | [37.8453; | 43.9805] | 0 | 0 | 0.41 | 0.00% |  |  |
| Tertile NA | 9 | 13.8422 | [ 5.7128; | 33.5403] | 1.8337 | 1.3542 | 15355.31 | 99.90% |  |  |

Note. k: number of sub-samples; mean: pooled mean; P^a^_sub_: p-value of the difference in mean values between all the sub-groups; P^b^_sub_: p-value of the difference in mean values between the sub-groups, without taking into account “NA” category

Table 9. Meta-analysis for PedMIDAS data.

| Sub-group | k | mean |  | 95%-CI | tau^2 | tau | Q | I^2 | P^a^_sub_ | P^b^_sub_ |
| --- | --- | --- | --- | --- | --- | --- | --- | --- | --- | --- |
| HA type |  |  |  |  |  |  |  |  | 0.0220 | 0.0366 |
| Migraine | 16 | 32.3009 | [26.1478; | 39.9018] | 0.1755 | 0.4189 | 568.92 | 97.40% |  |  |
| Migraine+TTH | 3 | 11.9041 | [ 4.7833; | 29.6257] | 0.6339 | 0.7962 | 105.95 | 98.10% |  |  |
| TACs | 0 | NA |  |  | -- | -- | -- | -- |  |  |
| TTH | 0 | NA |  |  | -- | -- | -- | -- |  |  |
| Migraine+TACs | 0 | NA |  |  | -- | -- | -- | -- |  |  |
| no HA type data | 1 | 40.51 | [32.5335; | 50.4421] | -- | -- | 0 | -- |  |  |
| Tertiles of F% |  |  |  |  |  |  |  |  | <0.0001 | <0.0001 |
| Tertile 1 | 15 | 26.3918 | [19.6732; | 35.4050] | 0.3286 | 0.5732 | 1111.01 | 98.70% |  |  |
| Tertile 2 | 1 | 39.6 | [29.8456; | 52.5424] | -- | -- | 0 | -- |  |  |
| Tertile 3 | 2 | 62.0401 | [56.3268; | 68.3328] | 0 | 0 | 0.24 | 0.00% |  |  |
| Tertile NA | 2 | 16.1307 | [10.8609; | 23.9576] | 0.0551 | 0.2347 | 3.03 | 67.00% |  |  |
| Age |  |  |  |  |  |  |  |  | -- | -- |
| Minors | 20 | 27.9874 | [21.4643; | 36.4930] | 0.355 | 0.5958 | 1258.46 | 98.50% |  |  |
| Adults | 0 | NA |  |  | -- | -- | -- | -- |  |  |
| NA | 0 | NA |  |  | -- | -- | -- | -- |  |  |
| Tertiles of Medication intake |  |  |  |  |  |  |  |  | <0.0001 | <0.0001 |
| Tertile 1 | 1 | 67.3 | [47.8718; | 94.6130] | -- | -- | 0 | -- |  |  |
| Tertile 2 | 2 | 32.3147 | [30.3489; | 34.4079] | 0 | 0 | 0.7 | 0.00% |  |  |
| Tertile 3 | 0 | NA |  |  | -- | -- | -- | -- |  |  |
| Tertile NA | 17 | 26.1816 | [19.4890; | 35.1725] | 0.3739 | 0.6115 | 1203.64 | 98.70% |  |  |

Note. k: number of sub-samples; mean: pooled mean; P^a^_sub_: p-value of the difference in mean values between all the sub-groups; P^b^_sub_: p-value of the difference in mean values between the sub-groups, without taking into account “NA” category

Table 10. Meta-analysis for HDI-E data.

| Sub-group | k | mean |  | 95%-CI | tau^2 | tau | Q | I^2 | P^a^_sub_ | P^b^_sub_ |
| --- | --- | --- | --- | --- | --- | --- | --- | --- | --- | --- |
| HA type |  |  |  |  |  |  |  |  | <0.0001 | -- |
| Migraine | 1 | 27 | [24.1132; | 30.2324] | -- | -- | 0 | -- |  |  |
| Migraine+TTH | 0 | NA |  |  | -- | -- | -- | -- |  |  |
| TACs | 0 | NA |  |  | -- | -- | -- | -- |  |  |
| TTH | 15 | 19.6379 | [18.2342; | 21.1497] | 0.016 | 0.1266 | 52.75 | 73.50% |  |  |
| Migraine+TACs | 0 | NA |  |  | -- | -- | -- | -- |  |  |
| no HA type data | 0 | NA |  |  | -- | -- | -- | -- |  |  |
| Tertiles of F% |  |  |  |  |  |  |  |  | 0.3372 | 0.2705 |
| Tertile 1 | 9 | 18.9529 | [17.1362; | 20.9623] | 0.0178 | 0.1336 | 31.12 | 74.30% |  |  |
| Tertile 2 | 0 | NA |  |  | -- | -- | -- | -- |  |  |
| Tertile 3 | 4 | 20.9546 | [18.0821; | 24.2833] | 0.0185 | 0.1361 | 14.94 | 79.90% |  |  |
| Tertile NA | 3 | 22.1254 | [17.5592; | 27.8790] | 0.0364 | 0.1908 | 15.3 | 86.90% |  |  |
| Age |  |  |  |  |  |  |  |  | -- | -- |
| Minors | 0 | NA |  |  | -- | -- | -- | -- |  |  |
| Adults | 16 | 20.0454 | [18.4813; | 21.7419] | 0.0221 | 0.1487 | 79.55 | 81.10% |  |  |
| NA | 0 | NA |  |  | -- | -- | -- | -- |  |  |
| Tertiles of Medication intake |  |  |  |  |  |  |  |  | -- | -- |
| Tertile 1 | 0 | NA |  |  | -- | -- | -- | -- |  |  |
| Tertile 2 | 0 | NA |  |  | -- | -- | -- | -- |  |  |
| Tertile 3 | 0 | NA |  |  | -- | -- | -- | -- |  |  |
| Tertile NA | 16 | 20.0454 | [18.4813; | 21.7419] | 0.0221 | 0.1487 | 79.55 | 81.10% |  |  |

Note. k: number of sub-samples; mean: pooled mean; P^a^_sub_: p-value of the difference in mean values between all the sub-groups; P^b^_sub_: p-value of the difference in mean values between the sub-groups, without taking into account “NA” category

Table 11. Meta-analysis for HDI-P data.

| Sub-group | k | mean |  | 95%-CI | tau^2 | tau | Q | I^2 | P^a^_sub_ | P^b^_sub_ |
| --- | --- | --- | --- | --- | --- | --- | --- | --- | --- | --- |
| HA type |  |  |  |  |  |  |  |  | <0.0001 | -- |
| Migraine | 1 | 34.7 | [32.1763; | 37.4216] | -- | -- | 0 | -- |  |  |
| Migraine+TTH | 0 | NA |  |  | -- | -- | -- | -- |  |  |
| TACs | 0 | NA |  |  | -- | -- | -- | -- |  |  |
| TTH | 15 | 23.275 | [22.3856; | 24.1997] | 0.0028 | 0.053 | 27.91 | 49.80% |  |  |
| Migraine+TACs | 0 | NA |  |  | -- | -- | -- | -- |  |  |
| no HA type data | 0 | NA |  |  | -- | -- | -- | -- |  |  |
| Tertiles of F% |  |  |  |  |  |  |  |  | 0.4401 | 0.3906 |
| Tertile 1 | 9 | 22.9454 | [21.6785; | 24.2864] | 0.0043 | 0.0656 | 18.22 | 56.10% |  |  |
| Tertile 2 | 0 | NA |  |  | -- | -- | -- | -- |  |  |
| Tertile 3 | 4 | 23.9776 | [22.0709; | 26.0491] | 0.0043 | 0.0654 | 7.23 | 58.50% |  |  |
| Tertile NA | 3 | 26.6082 | [20.2671; | 34.9332] | 0.055 | 0.2346 | 49.96 | 96.00% |  |  |
| Age |  |  |  |  |  |  |  |  | -- | -- |
| Minors | 0 | NA |  |  | -- | -- | -- | -- |  |  |
| Adults | 16 | 23.9128 | [22.3507; | 25.5841] | 0.0158 | 0.1257 | 123.66 | 87.90% |  |  |
| NA | 0 | NA |  |  | -- | -- | -- | -- |  |  |
| Tertiles of Medication intake |  |  |  |  |  |  |  |  | -- | -- |
| Tertile 1 | 0 | NA |  |  | -- | -- | -- | -- |  |  |
| Tertile 2 | 0 | NA |  |  | -- | -- | -- | -- |  |  |
| Tertile 3 | 0 | NA |  |  | -- | -- | -- | -- |  |  |
| Tertile NA | 16 | 23.9128 | [22.3507; | 25.5841] | 0.0158 | 0.1257 | 123.66 | 87.90% |  |  |

Note. k: number of sub-samples; mean: pooled mean; P^a^_sub_: p-value of the difference in mean values between all the sub-groups; P^b^_sub_: p-value of the difference in mean values between the sub-groups, without taking into account “NA” category

Supplementary Figure 1. MIDAS-built coefficient, differences based on females’ percentage tertiles

Supplementary Figure 2. MIDAS-built coefficient, differences based on tertiles of age

Supplementary Figure 3. MIDAS-built coefficient, differences based on primary headache type

Supplementary Figure 4. MIDAS-built coefficient, differences based on medications’ intake tertiles

Supplementary Figure 5. HIT-6-built coefficient, differences based on females’ percentage tertiles

Supplementary Figure 6. HIT-6-built coefficient, differences based on tertiles of age

Supplementary Figure 7. HIT-6-built coefficient, differences based on primary headache type

Supplementary Figure 8. HIT-6-built coefficient, differences based on medications’ intake tertiles

Supplementary Figure 9. MIDAS & HIT-6-built coefficient, differences based on females’ percentage tertiles

Supplementary Figure 10. MIDAS & HIT-6-built coefficient, differences based on tertiles of age

Supplementary Figure 11. MIDAS & HIT-6-built coefficient, differences based on primary headache type

Supplementary Figure 12. MIDAS & HIT-6-built coefficient, differences based on medications’ intake tertiles
