# Supplementary material for: The impact of primary headaches on disability outcomes: a literature review and meta-analysis to inform future iterations of the Global Burden of Disease study
Source: J Headache Pain. 2024 Mar 4;25(1):27. doi: 10.1186/s10194-024-01735-0 (PMC10910736; doi:10.1186/s10194-024-01735-0)
Supplement: Supplementary file 2 — Supplementary Material 2. [file 10194_2024_1735_MOESM2_ESM.docx]

**Migraine**

1. Straube A, Broessner G, Gaul C, et al. Real-world effectiveness of fremanezumab in patients with migraine switching from another mAb targeting the CGRP pathway: a subgroup analysis of the Finesse Study. J Headache Pain. 2023;24(1):59. Published 2023 May 23. doi:10.1186/s10194-023-01593-2
2. Ashina M, Roos C, Li LQ, et al. Long-term treatment with lasmiditan in patients with migraine: Results from the open-label extension of the CENTURION randomized trial. Cephalalgia. 2023;43(4):3331024231161745. doi:10.1177/03331024231161745
3. Barbanti P, Egeo G, Aurilia C, et al. Early and sustained efficacy of fremanezumab over 24-weeks in migraine patients with multiple preventive treatment failures: the multicenter, prospective, real-life FRIEND2 study. J Headache Pain. 2023;24(1):30. Published 2023 Mar 23. doi:10.1186/s10194-023-01561-w
4. Russo CV, Saccà F, Braca S, et al. Anti-calcitonin gene-related peptide monoclonal antibodies for the treatment of vestibular migraine: A prospective observational cohort study. *Cephalalgia*. 2023;43(4):3331024231161809. doi:10.1177/03331024231161809
5. Iannone LF, Burgalassi A, Vigani G, et al. Switching anti-CGRP(R) monoclonal antibodies in multi-assessed non-responder patients and implications for ineffectiveness criteria: A retrospective cohort study. *Cephalalgia*. 2023;43(4):3331024231160519. doi:10.1177/03331024231160519
6. Argyriou AA, Dermitzakis EV, Xiromerisiou G, et al. Efficacy and safety of fremanezumab for migraine prophylaxis in patients with at least three previous preventive failures: Prospective, multicenter, real-world data from a Greek registry. *Eur J Neurol*. 2023;30(5):1435-1442. doi:10.1111/ene.15740
7. Underwood M, Achana F, Carnes D, et al. Supportive Self-Management Program for People With Chronic Headaches and Migraine: A Randomized Controlled Trial and Economic Evaluation. *Neurology*. 2023;100(13):e1339-e1352. doi:10.1212/WNL.0000000000201518
8. Messina R, Cetta I, Colombo B, Filippi M. Tracking the evolution of non-headache symptoms through the migraine attack. *J Headache Pain*. 2022;23(1):149. Published 2022 Nov 23. doi:10.1186/s10194-022-01525-6
9. Gantenbein AR, Agosti R, Kamm CP, et al. Swiss QUality of life and healthcare impact Assessment in a Real-world Erenumab treated migraine population (SQUARE study): interim results [published correction appears in J Headache Pain. 2022 Dec 19;23(1):161]. *J Headache Pain*. 2022;23(1):142. Published 2022 Nov 18. doi:10.1186/s10194-022-01515-8
10. Lipton RB, Pozo-Rosich P, Blumenfeld AM, et al. Effect of Atogepant for Preventive Migraine Treatment on Patient-Reported Outcomes in the Randomized, Double-blind, Phase 3 ADVANCE Trial. *Neurology*. 2023;100(8):e764-e777. doi:10.1212/WNL.0000000000201568
11. Seng EK, Shapiro RE, Buse DC, Robbins MS, Lipton RB, Parker A. The unique role of stigma in migraine-related disability and quality of life. *Headache*. 2022;62(10):1354-1364. doi:10.1111/head.14401
12. Cantarelli L, Pestana Grafiña D, Gonzalez Perez A, et al. Efficacy and Safety of Erenumab, Galcanezumab, and Fremanezumab in the Treatment of Drug-Resistant Chronic Migraine: Experience in Real Clinical Practice. *Ann Pharmacother*. 2023;57(4):416-424. doi:10.1177/10600280221118402
13. Ailani J, Andrews JS, Tockhorn-Heidenreich A, Wenzel R, Rettiganti M. Effect of Galcanezumab on Total Pain Burden in Patients Who Had Previously Not Benefited from Migraine Preventive Medication (CONQUER Trial): A Post Hoc Analysis. *Adv Ther*. 2022;39(10):4544-4555. doi:10.1007/s12325-022-02233-y
14. Hashimoto Y, Komori M, Tanji Y, Ozeki A, Hirata K. Lasmiditan for single migraine attack in Japanese patients with cardiovascular risk factors: subgroup analysis of a phase 2 randomized placebo-controlled trial. *Expert Opin Drug Saf*. 2022;21(12):1495-1503. doi:10.1080/14740338.2022.2078302
15. Soleimanian-Boroujeni F, Badihian N, Badihian S, Shaygannejad V, Gorji Y. The efficacy of transdiagnostic cognitive behavioral therapy on migraine headache: a pilot, feasibility study. *BMC Neurol*. 2022;22(1):230. Published 2022 Jun 22. doi:10.1186/s12883-022-02729-8
16. Silvestro M, Tessitore A, Orologio I, et al. Galcanezumab effect on "whole pain burden" and multidimensional outcomes in migraine patients with previous unsuccessful treatments: a real-world experience. *J Headache Pain*. 2022;23(1):69. Published 2022 Jun 13. doi:10.1186/s10194-022-01436-6
17. Lloyd JO, Hill B, Murphy M, Al-Kaisy A, Andreou AP, Lambru G. Single-Pulse Transcranial Magnetic Stimulation for the preventive treatment of difficult-to-treat migraine: a 12-month prospective analysis. *J Headache Pain*. 2022;23(1):63. Published 2022 Jun 6. doi:10.1186/s10194-022-01428-6
18. Schiano di Cola F, Caratozzolo S, Bolchini M, et al. CGRP-monoclonal antibodies in difficult-to-treat chronic migraine patients. *Neurol Sci*. 2022;43(9):5763-5764. doi:10.1007/s10072-022-06154-0
19. Khalil M, Moreno-Ajona D, Villar-Martínez MD, Greenwood F, Hoffmann J, Goadsby PJ. Erenumab in chronic migraine: Experience from a UK tertiary centre and comparison with other real-world evidence. *Eur J Neurol*. 2022;29(8):2473-2480. doi:10.1111/ene.15364
20. Ornello R, Baraldi C, Guerzoni S, et al. Comparing the relative and absolute effect of erenumab: is a 50% response enough? Results from the ESTEEMen study. *J Headache Pain*. 2022;23(1):38. Published 2022 Mar 19. doi:10.1186/s10194-022-01408-w
21. Lampl C, Rapoport AM, Cohen JM, et al. Efficacy and quality-of-life improvements with fremanezumab treatment in patients with difficult-to-treat migraine with associated neurological dysfunction. *Eur J Neurol*. 2022;29(7):2129-2137. doi:10.1111/ene.15328
22. Kwon S, Gil YE, Lee MJ. Real-world efficacy of galcanezumab for the treatment of migraine in Korean patients. *Cephalalgia*. 2022;42(8):705-714. doi:10.1177/03331024221076481
23. Liang Z, Thomas L, Jull G, Treleaven J. The Neck Disability Index Reflects Allodynia and Headache Disability but Not Cervical Musculoskeletal Dysfunction in Migraine. *Phys Ther*. 2022;102(5):pzac027. doi:10.1093/ptj/pzac027
24. Lotfi K, Askari G, Mohammad H, Fadel A, Khorvash F, Arab A. Association between dietary acid load and clinical features of migraine headaches among Iranian individuals. *Sci Rep*. 2022;12(1):2460. Published 2022 Feb 14. doi:10.1038/s41598-022-06515-x
25. Hedayat M, Nazarbaghi S, Heidari M, Sharifi H. Venlafaxine can reduce the migraine attacks as well as amitriptyline: A noninferiority randomized trial. *Clin Neurol Neurosurg*. 2022;214:107151. doi:10.1016/j.clineuro.2022.107151
26. Granato A, Furlanis G, D'Acunto L, Olivo S, Buoite Stella A, Manganotti P. Lifestyle impact on migraine during home confinement. *Acta Neurol Belg*. 2022;122(2):497-503. doi:10.1007/s13760-021-01856-2
27. Iannone LF, Fattori D, Benemei S, Chiarugi A, Geppetti P, De Cesaris F. Long-Term Effectiveness of Three Anti-CGRP Monoclonal Antibodies in Resistant Chronic Migraine Patients Based on the MIDAS score. *CNS Drugs*. 2022;36(2):191-202. doi:10.1007/s40263-021-00893-y
28. McAllister P, Winner PK, Ailani J, et al. Eptinezumab treatment initiated during a migraine attack is associated with meaningful improvement in patient-reported outcome measures: secondary results from the randomized controlled RELIEF study. *J Headache Pain*. 2022;23(1):22. Published 2022 Feb 7. doi:10.1186/s10194-021-01376-7
29. Iannone LF, Fattori D, Benemei S, Chiarugi A, Geppetti P, De Cesaris F. Predictors of sustained response and effects of the discontinuation of anti-calcitonin gene related peptide antibodies and reinitiation in resistant chronic migraine. *Eur J Neurol*. 2022;29(5):1505-1513. doi:10.1111/ene.15260
30. Al-Kaisy A, Palmisani S, Carganillo R, et al. Safety and Efficacy of 10 kHz Spinal Cord Stimulation for the Treatment of Refractory Chronic Migraine: A Prospective Long-Term Open-Label Study. *Neuromodulation*. 2022;25(1):103-113. doi:10.1111/ner.13465
31. Guerzoni S, Baraldi C, Pensato U, et al. Chronic migraine evolution after 3 months from erenumab suspension: real-world-evidence-life data. *Neurol Sci*. 2022;43(6):3823-3830. doi:10.1007/s10072-022-05870-x
32. Zecca C, Cargnin S, Schankin C, et al. Clinic and genetic predictors in response to erenumab. *Eur J Neurol*. 2022;29(4):1209-1217. doi:10.1111/ene.15236
33. Alpuente A, Gallardo VJ, Caronna E, Torres-Ferrus M, Pozo-Rosich P. In search of a gold standard patient-reported outcome measure to use in the evaluation and treatment-decision making in migraine prevention. A real-world evidence study. *J Headache Pain*. 2021;22(1):151. Published 2021 Dec 13. doi:10.1186/s10194-021-01366-9
34. Vernieri F, Altamura C, Brunelli N, et al. Rapid response to galcanezumab and predictive factors in chronic migraine patients: A 3-month observational, longitudinal, cohort, multicenter, Italian real-life study [published correction appears in Eur J Neurol. 2023 Jun;30(6):1841]. *Eur J Neurol*. 2022;29(4):1198-1208. doi:10.1111/ene.15197
35. Pinheiro CF, Bevilaqua-Grossi D, Florencio LL, et al. Is kinesiophobia related to fear of falling, dizziness disability, and migraine disability in patients with migraine?. *Physiother Theory Pract*. 2022;38(13):2727-2735. doi:10.1080/09593985.2021.1996496
36. Ramsden CE, Zamora D, Faurot KR, et al. Dietary alteration of n-3 and n-6 fatty acids for headache reduction in adults with migraine: randomized controlled trial. *BMJ*. 2021;374:n1448. Published 2021 Jun 30. doi:10.1136/bmj.n1448
37. Surani MK, Yousuf M, Anjum N, Khan S, Hasan G, Hussain S. Topiramate For Migraine Prophylaxis Among Children Aged 5 To 15 Years. *J Ayub Med Coll Abbottabad*. 2021;33(3):480-483.
38. Martinelli D, Arceri S, De Icco R, et al. BoNT-A efficacy in high frequency migraine: an open label, single arm, exploratory study applying the PREEMPT paradigm. *Cephalalgia*. 2022;42(2):170-175. doi:10.1177/03331024211034508
39. Hamamci M, Songur MS, Aslan Bayhan S, Bayhan HA. Is ocular vascularity affected in young migraine patients? A pilot study. *J Clin Neurosci*. 2021;91:144-151. doi:10.1016/j.jocn.2021.06.045
40. Florencio LL, de Oliveira AS, Pinheiro CF, et al. Comparison of cervical muscle isometric force between migraine subgroups or migraine-associated neck pain: a controlled study. *Sci Rep*. 2021;11(1):15434. Published 2021 Jul 29. doi:10.1038/s41598-021-95078-4
41. Barbanti P, Aurilia C, Cevoli S, et al. Long-term (48 weeks) effectiveness, safety, and tolerability of erenumab in the prevention of high-frequency episodic and chronic migraine in a real world: Results of the EARLY 2 study. *Headache*. 2021;61(9):1351-1363. doi:10.1111/head.14194
42. Moraes Alves AL, Silva IK, Paula Lemos PH, Lomachinsky Torres V, Crevanzi Arraes E, Sampaio Rocha-Filho PA. FRAMES protocol versus simple advice for medication-overuse headache: a prospective, randomized, controlled clinical trial. *Acta Neurol Belg*. 2021;121(5):1259-1264. doi:10.1007/s13760-021-01758-3
43. Eghtesadi M, Leroux E, Pagé G. Real-Life Response to Erenumab in a Therapy-Resistant Case Series of Migraine Patients From the Province of Québec, Eastern Canada. *Clin Drug Investig*. 2021;41(8):733-739. doi:10.1007/s40261-021-01059-w
44. Baraldi C, Castro FL, Cainazzo MM, Pani L, Guerzoni S. Predictors of response to erenumab after 12 months of treatment. *Brain Behav*. 2021;11(8):e2260. doi:10.1002/brb3.2260
45. Di Antonio S, Castaldo M, Ponzano M, et al. Disability, burden, and symptoms related to sensitization in migraine patients associate with headache frequency. *Scand J Pain*. 2021;21(4):766-777. Published 2021 Jul 13. doi:10.1515/sjpain-2021-0050
46. Powers SW, Coffey CS, Chamberlin LA, et al. Prevalence of Headache Days and Disability 3 Years After Participation in the Childhood and Adolescent Migraine Prevention Medication Trial. *JAMA Netw Open*. 2021;4(7):e2114712. Published 2021 Jul 1. doi:10.1001/jamanetworkopen.2021.14712
47. Barbanti P, Fofi L, Grazzi L, et al. Clinical features, disease progression, and use of healthcare resources in a large sample of 866 patients from 24 headache centers: A real-life perspective from the Italian chROnic migraiNe (IRON) project. *Headache*. 2021;61(6):936-950. doi:10.1111/head.14123
48. D'Amico D, Grazzi L, Guastafierro E, Sansone E, Leonardi M, Raggi A. Withdrawal failure in patients with chronic migraine and medication overuse headache. *Acta Neurol Scand*. 2021;144(4):408-417. doi:10.1111/ane.13475
49. Kanar HS, Toz HT, Penbe A. Comparison of retinal nerve fiber layer, macular ganglion cell complex and choroidal thickness in patients with migraine with and without aura by using optical coherence tomography. *Photodiagnosis Photodyn Ther*. 2021;34:102323. doi:10.1016/j.pdpdt.2021.102323
50. Lipton RB, Cohen JM, Galic M, et al. Effects of fremanezumab in patients with chronic migraine and comorbid depression: Subgroup analysis of the randomized HALO CM study. *Headache*. 2021;61(4):662-672. doi:10.1111/head.14097
51. Amoozegar F, Khan Z, Oviedo-Ovando M, Sauriol S, Rochdi D. The Burden of Illness of Migraine in Canada: New Insights on Humanistic and Economic Cost. *Can J Neurol Sci*. 2022;49(2):249-262. doi:10.1017/cjn.2021.75
52. Kudrow D, Cady RK, Allan B, et al. Long-term safety and tolerability of eptinezumab in patients with chronic migraine: a 2-year, open-label, phase 3 trial. *BMC Neurol*. 2021;21(1):126. Published 2021 Mar 19. doi:10.1186/s12883-021-02123-w
53. Liu YF, Dornhoffer JR, Donaldson L, Rizk HG. Impact of caloric test asymmetry on response to treatment in vestibular migraine. *J Laryngol Otol*. 2021;135(4):320-326. doi:10.1017/S0022215121000712
54. Cainazzo MM, Baraldi C, Ferrari A, Lo Castro F, Pani L, Guerzoni S. Erenumab for the preventive treatment of chronic migraine complicated with medication overuse headache: an observational, retrospective, 12-month real-life study. *Neurol Sci*. 2021;42(10):4193-4202. doi:10.1007/s10072-021-05105-5
55. Esmael A, Abdelsalam M, Shoukri A, Elsherif M. Subjective cognitive impairment in patients with transformed migraine and the associated psychological and sleep disturbances. *Sleep Breath*. 2021;25(4):2119-2126. doi:10.1007/s11325-021-02308-0
56. Speck RM, Yu R, Ford JH, Ayer DW, Bhandari R, Wyrwich KW. Psychometric validation and meaningful within-patient change of the Migraine-Specific Quality of Life questionnaire version 2.1 electronic patient-reported outcome in patients with episodic and chronic migraine. *Headache*. 2021;61(3):511-526. doi:10.1111/head.14031
57. Lanteri-Minet M, Goadsby PJ, Reuter U, et al. Effect of erenumab on functional outcomes in patients with episodic migraine in whom 2-4 preventives were not useful: results from the LIBERTY study. *J Neurol Neurosurg Psychiatry*. 2021;92(5):466-472. doi:10.1136/jnnp-2020-324396
58. Ashina M, Goadsby PJ, Reuter U, et al. Long-term efficacy and safety of erenumab in migraine prevention: Results from a 5-year, open-label treatment phase of a randomized clinical trial. *Eur J Neurol*. 2021;28(5):1716-1725. doi:10.1111/ene.14715
59. Barbanti P, Aurilia C, Egeo G, et al. Erenumab in the prevention of high-frequency episodic and chronic migraine: Erenumab in Real Life in Italy (EARLY), the first Italian multicenter, prospective real-life study. *Headache*. 2021;61(2):363-372. doi:10.1111/head.14032
60. Polk AN, Protti TA, Smitherman TA. Allodynia and Disability in Migraine: The Mediating Role of Stress. *Headache*. 2020;60(10):2281-2290. doi:10.1111/head.14012
61. Shah S, Calderon MD, Crain N, Pham J, Rinehart J. Effectiveness of onabotulinumtoxinA (BOTOX) in pediatric patients experiencing migraines: a randomized, double-blinded, placebo-controlled crossover study in the pediatric pain population. *Reg Anesth Pain Med*. 2021;46(1):41-48. doi:10.1136/rapm-2020-101605
62. Houts CR, McGinley JS, Wirth RJ, Cady R, Lipton RB. Reliability and validity of the 6-item Headache Impact Test in chronic migraine from the PROMISE-2 study. *Qual Life Res*. 2021;30(3):931-943. doi:10.1007/s11136-020-02668-2
63. Ailani J, Andrews JS, Rettiganti M, Nicholson RA. Impact of galcanezumab on total pain burden: findings from phase 3 randomized, double-blind, placebo-controlled studies in patients with episodic or chronic migraine (EVOLVE-1, EVOLVE-2, and REGAIN trials). *J Headache Pain*. 2020;21(1):123. Published 2020 Oct 17. doi:10.1186/s10194-020-01190-7
64. Altamura C, Cevoli S, Aurilia C, et al. Locking down the CGRP pathway during the COVID-19 pandemic lockdown: the PandeMig study. *Neurol Sci*. 2020;41(12):3385-3389. doi:10.1007/s10072-020-04767-x
65. Ford J, Tassorelli C, Leroux E, et al. Changes in patient functioning and disability: results from a phase 3, double-blind, randomized, placebo-controlled clinical trial evaluating galcanezumab for chronic migraine prevention (REGAIN). *Qual Life Res*. 2021;30(1):105-115. doi:10.1007/s11136-020-02623-1
66. Qi Y, Zhang Y, Luo X, et al. Efficacy of patent foramen ovale closure for treating migraine: a prospective follow-up study. *J Investig Med*. 2021;69(1):7-12. doi:10.1136/jim-2020-001323
67. Goadsby PJ, Silberstein SD, Yeung PP, et al. Long-term safety, tolerability, and efficacy of fremanezumab in migraine: A randomized study. *Neurology*. 2020;95(18):e2487-e2499. doi:10.1212/WNL.0000000000010600
68. Rodríguez-Almagro D, Achalandabaso-Ochoa A, Obrero-Gaitán E, Osuna-Pérez MC, Ibáñez-Vera AJ, Lomas-Vega R. Sleep Alterations in Female College Students with Migraines. *Int J Environ Res Public Health*. 2020;17(15):5456. Published 2020 Jul 29. doi:10.3390/ijerph17155456
69. Pearl TA, Dumkrieger G, Chong CD, Dodick DW, Schwedt TJ. Impact of Depression and Anxiety Symptoms on Patient-Reported Outcomes in Patients With Migraine: Results From the American Registry for Migraine Research (ARMR). *Headache*. 2020;60(9):1910-1919. doi:10.1111/head.13911
70. Zhang Y, Xu T, Wang Z, et al. Differences in topological properties of functional brain networks between menstrually-related and non-menstrual migraine without aura. *Brain Imaging Behav*. 2021;15(3):1450-1459. doi:10.1007/s11682-020-00344-0
71. Lambru G, Hill B, Murphy M, Tylova I, Andreou AP. A prospective real-world analysis of erenumab in refractory chronic migraine. *J Headache Pain*. 2020;21(1):61. Published 2020 Jun 1. doi:10.1186/s10194-020-01127-0
72. Altamura C, Cecchi G, Bravo M, et al. The Healthy Eating Plate Advice for Migraine Prevention: An Interventional Study. *Nutrients*. 2020;12(6):1579. Published 2020 May 28. doi:10.3390/nu12061579
73. Houle M, Marchand AA, Descarreaux M. Can Headache Profile Predict Future Disability: A Cohort Study. *Clin J Pain*. 2020;36(8):594-600. doi:10.1097/AJP.0000000000000843
74. Qin Z, Su J, He XW, et al. Disrupted functional connectivity between sub-regions in the sensorimotor areas and cortex in migraine without aura. *J Headache Pain*. 2020;21(1):47. Published 2020 May 6. doi:10.1186/s10194-020-01118-1
75. Deng Y, Zheng M, He L, Yang J, Yu G, Wang J. A Head-to-Head Comparison of Percutaneous Mastoid Electrical Stimulator and Supraorbital Transcutaneous Stimulator in the Prevention of Migraine: A Prospective, Randomized Controlled Study. *Neuromodulation*. 2020;23(6):770-777. doi:10.1111/ner.13127
76. Lipton RB, Lombard L, Ruff DD, et al. Trajectory of migraine-related disability following long-term treatment with lasmiditan: results of the GLADIATOR study. *J Headache Pain*. 2020;21(1):20. Published 2020 Feb 24. doi:10.1186/s10194-020-01088-4
77. Ulusoy EK, Bolattürk ÖF. The effect of greater occipital nerve blockade on the quality of life, disability and comorbid depression, anxiety, and sleep disturbance in patients with chronic migraine. *Neurol Sci*. 2020;41(7):1829-1835. doi:10.1007/s10072-020-04286-9
78. Togha M, Haghdoost F, Khorsha F, Razeghi Jahromi S, Ghorbani Z. Body Mass Index and its Association with Migraine Characteristics in Female Patients. *Arch Iran Med*. 2019;22(10):554-559. Published 2019 Oct 1.
79. Seng EK, Singer AB, Metts C, et al. Does Mindfulness-Based Cognitive Therapy for Migraine Reduce Migraine-Related Disability in People with Episodic and Chronic Migraine? A Phase 2b Pilot Randomized Clinical Trial. *Headache*. 2019;59(9):1448-1467. doi:10.1111/head.13657
80. Ashina M, Vasudeva R, Jin L, et al. Onset of Efficacy Following Oral Treatment With Lasmiditan for the Acute Treatment of Migraine: Integrated Results From 2 Randomized Double-Blind Placebo-Controlled Phase 3 Clinical Studies. *Headache*. 2019;59(10):1788-1801. doi:10.1111/head.13636
81. Vernieri F, Paolucci M, Altamura C, et al. Onabotulinumtoxin-A in Chronic Migraine: Should Timing and Definition of Non-Responder Status Be Revised? Suggestions From a Real-Life Italian Multicenter Experience. *Headache*. 2019;59(8):1300-1309. doi:10.1111/head.13617
82. Brandes JL, Klise S, Krege JH, et al. Interim results of a prospective, randomized, open-label, Phase 3 study of the long-term safety and efficacy of lasmiditan for acute treatment of migraine (the GLADIATOR study). *Cephalalgia*. 2019;39(11):1343-1357. doi:10.1177/0333102419864132
83. Silberstein SD, Stauffer VL, Day KA, Lipsius S, Wilson MC. Galcanezumab in episodic migraine: subgroup analyses of efficacy by high versus low frequency of migraine headaches in phase 3 studies (EVOLVE-1 & EVOLVE-2) [published correction appears in J Headache Pain. 2019 Dec 27;20(1):118]. *J Headache Pain*. 2019;20(1):75. Published 2019 Jun 28. doi:10.1186/s10194-019-1024-x
84. Onder H, Hamamci M, Alpua M, Ulusoy EK. Comorbid fibromyalgia in migraine patients: clinical significance and impact on daily life. *Neurol Res*. 2019;41(10):909-915. doi:10.1080/01616412.2019.1630164
85. Dehghani A, Karatas H. Mouse Models of Familial Hemiplegic Migraine for Studying Migraine Pathophysiology. *Curr Neuropharmacol*. 2019;17(10):961-973. doi:10.2174/1570159X17666190513085013
86. Young WB, Ivan Lopez J, Rothrock JF, et al. Effects of onabotulinumtoxinA treatment in chronic migraine patients with and without daily headache at baseline: results from the COMPEL Study. *J Headache Pain*. 2019;20(1):12. Published 2019 Feb 1. doi:10.1186/s10194-018-0953-0
87. Ciere Y, Snippe E, Padberg M, et al. The role of state and trait positive affect and mindfulness in affective reactivity to pain in chronic migraine. *Health Psychol*. 2019;38(1):94-102. doi:10.1037/hea0000692
88. Granato A, Fantini J, Monti F, et al. Dramatic placebo effect of high frequency repetitive TMS in treatment of chronic migraine and medication overuse headache. *J Clin Neurosci*. 2019;60:96-100. doi:10.1016/j.jocn.2018.09.021
89. Raggi A, Covelli V, Guastafierro E, et al. Validation of a self-reported instrument to assess work-related difficulties in patients with migraine: the HEADWORK questionnaire. *J Headache Pain*. 2018;19(1):85. Published 2018 Sep 10. doi:10.1186/s10194-018-0914-7
90. D'Amico D, Sansone E, Grazzi L, et al. Multimorbidity in patients with chronic migraine and medication overuse headache. *Acta Neurol Scand*. 2018;138(6):515-522. doi:10.1111/ane.13014
91. Kucukdurmaz F, Inanc Y, Inanc Y, Resim S. Sexual dysfunction and distress in premenopausal women with migraine: association with depression, anxiety and migraine-related disability. *Int J Impot Res*. 2018;30(5):265-271. doi:10.1038/s41443-018-0049-z
92. Yalinay Dikmen P, Kosak S, Ilgaz Aydinlar E, Sagduyu Kocaman A. A single-center retrospective study of onabotulinumtoxinA for treatment of 245 chronic migraine patients: survey results of a real-world experience. *Acta Neurol Belg*. 2018;118(3):475-484. doi:10.1007/s13760-018-0978-9
93. Tomé-Pires C, Solé E, Racine M, et al. The relative importance of anxiety and depression in pain impact in individuals with migraine headaches. *Scand J Pain*. 2016;13:109-113. doi:10.1016/j.sjpain.2016.08.002
94. Ashina M, Dodick D, Goadsby PJ, et al. Erenumab (AMG 334) in episodic migraine: Interim analysis of an ongoing open-label study. *Neurology*. 2017;89(12):1237-1243. doi:10.1212/WNL.0000000000004391
95. Lee SH, Kang Y, Cho SJ. Subjective cognitive decline in patients with migraine and its relationship with depression, anxiety, and sleep quality. *J Headache Pain*. 2017;18(1):77. doi:10.1186/s10194-017-0779-1
96. Fernández-de-Las-Peñas C, Falla D, Palacios-Ceña M, et al. Perceived Pain Extent is Not Associated With Widespread Pressure Pain Sensitivity, Clinical Features, Related Disability, Anxiety, or Depression in Women With Episodic Migraine. *Clin J Pain*. 2018;34(3):217-221. doi:10.1097/AJP.0000000000000537
97. De Tommaso M, Sciruicchio V, Delussi M, et al. Symptoms of central sensitization and comorbidity for juvenile fibromyalgia in childhood migraine: an observational study in a tertiary headache center. *J Headache Pain*. 2017;18(1):59. doi:10.1186/s10194-017-0764-8
98. Lillis J, Graham Thomas J, Seng EK, et al. Importance of Pain Acceptance in Relation to Headache Disability and Pain Interference in Women With Migraine and Overweight/Obesity. *Headache*. 2017;57(5):709-718. doi:10.1111/head.13058
99. Santangelo G, Russo A, Trojano L, et al. Cognitive dysfunctions and psychological symptoms in migraine without aura: a cross-sectional study. *J Headache Pain*. 2016;17(1):76. doi:10.1186/s10194-016-0667-0
100. Song TJ, Cho SJ, Kim WJ, Yang KI, Yun CH, Chu MK. Anxiety and depression in probable migraine: A population-based study. *Cephalalgia*. 2017;37(9):845-854. doi:10.1177/0333102416653235
101. Russo A, Tessitore A, Conte F, Marcuccio L, Giordano A, Tedeschi G. Transcutaneous supraorbital neurostimulation in "de novo" patients with migraine without aura: the first Italian experience. *J Headache Pain*. 2015;16:69. doi:10.1186/s10194-015-0551-3
102. Straube A, Ellrich J, Eren O, Blum B, Ruscheweyh R. Treatment of chronic migraine with transcutaneous stimulation of the auricular branch of the vagal nerve (auricular t-VNS): a randomized, monocentric clinical trial. *J Headache Pain*. 2015;16:543. doi:10.1186/s10194-015-0543-3
103. Foote HW, Hamer JD, Roland MM, Landy SR, Smitherman TA. Psychological flexibility in migraine: A study of pain acceptance and values-based action. *Cephalalgia*. 2016;36(4):317-324. doi:10.1177/0333102415590238
104. Merki-Feld GS, Imthurn B, Langner R, Seifert B, Gantenbein AR. Positive effects of the progestin desogestrel 75 μg on migraine frequency and use of acute medication are sustained over a treatment period of 180 days. *J Headache Pain*. 2015;16:522. doi:10.1186/s10194-015-0522-8
105. Gaul C, Diener HC, Danesch U; Migravent® Study Group. Improvement of migraine symptoms with a proprietary supplement containing riboflavin, magnesium and Q10: a randomized, placebo-controlled, double-blind, multicenter trial. *J Headache Pain*. 2015;16:516. doi:10.1186/s10194-015-0516-6
106. Zhang N, Chen CF, Yu FY. Effects of pregabalin on central sensitization in patients with migraine. *Int J Clin Pharmacol Ther*. 2015;53(4):277-283. doi:10.5414/CP202205
107. Akdal G, Baykan B, Ertaş M, et al. Population-based study of vestibular symptoms in migraineurs. *Acta Otolaryngol*. 2015;135(5):435-439. doi:10.3109/00016489.2014.969382
108. Shaik MM, Hassan NB, Tan HL, Gan SH. Quality of life and migraine disability among female migraine patients in a tertiary hospital in Malaysia. *Biomed Res Int*. 2015;2015:523717. doi:10.1155/2015/523717
109. Zandifar A, Iraji N, Taheriun M, Tajaddini M, Javanmard SH. Association of the long pentraxin PTX3 gene polymorphism (rs3816527) with migraine in an Iranian population. *J Neurol Sci*. 2015;349(1-2):185-189. doi:10.1016/j.jns.2015.01.015
110. Adams AM, Serrano D, Buse DC, et al. The impact of chronic migraine: The Chronic Migraine Epidemiology and Outcomes (CaMEO) Study methods and baseline results. *Cephalalgia*. 2015;35(7):563-578. doi:10.1177/0333102414552532
111. Meise R, Carvalho GF, Thiel C, Luedtke K. Additional effects of pain neuroscience education combined with physiotherapy on the headache frequency of adult patients with migraine: A randomized controlled trial. Cephalalgia. 2023;43(2). doi:10.1177/03331024221144781
112. Grazzi L, Montisano DA, Rizzoli P, et al. A Single-Group Study on the Effect of OnabotulinumtoxinA in Patients with Chronic Migraine Associated with Medication Overuse Headache: Pain Catastrophizing Plays a Role. *Toxins (Basel)*. 2023;15(2):86. Published 2023 Jan 17. doi:10.3390/toxins15020086
113. Starling AJ, Cowan RP, Buse DC, et al. Eptinezumab improved patient-reported outcomes in patients with migraine and medication-overuse headache: Subgroup analysis of the randomized PROMISE-2 trial. *Headache*. 2023;63(2):264-274. doi:10.1111/head.14434
114. Zhou Y, Gong L, Yang Y, et al*.* Spatio-temporal dynamics of resting-state brain networks are associated with migraine disability. *J Headache Pain* 24, 13 (2023). <https://doi.org/10.1186/s10194-023-01551-y>
115. Wei HL, Hu TT, Wang JJ, Wang MY, Yu YS, Zhang H. Potential predictors for the efficacy of non-steroidal anti-inflammatory drugs in patients with migraine. *Saudi Pharm J*. 2023;31(5):692-697. doi:10.1016/j.jsps.2023.03.010
116. Tsai MC, Tsai CL, Liang CS, et al. Identification of genetic risk loci for depression and migraine comorbidity in Han Chinese residing in Taiwan. *Front Psychiatry*. 2023;13:1067503. Published 2023 Jan 10. doi:10.3389/fpsyt.2022.1067503
117. Kyung-Hee Cho, Kyungmi Oh, et al. Relationships among Symptoms, Disability, Type D Personality, and Quality of Life in Patients with Migraine: A Cross-Sectional Study in South Korea. *Pain Management Nursing*. 2023 180 doi.org/10.1016/j.pmn.2022.08.001.
118. di Cola FS, Bolchini M, Caratozzolo S, et al. Migraine Disability Improvement during Treatment with Galcanezumab in Patients with Chronic and High Frequency Episodic Migraine. *Neurol Int*. 2023;15(1):273-284. Published 2023 Feb 13. doi:10.3390/neurolint15010017
119. Grazzi L, Montisano DA, Raggi A, Rizzoli P. The Be-Home Kids Program: An Integrated Approach for Delivering Behavioral Therapies to Adolescents with Episodic and Chronic Migraine. *Brain Sci*. 2023;13(4):699. Published 2023 Apr 21. doi:10.3390/brainsci13040699
120. Altamura C, Brunelli N, Viticchi G, et al. Quantitative and Qualitative Pain Evaluation in Response to OnabotulinumtoxinA for Chronic Migraine: An Observational Real-Life Study. *Toxins (Basel)*. 2023;15(4):284. Published 2023 Apr 15. doi:10.3390/toxins15040284
121. Ezzati A, Fanning KM, Reed ML, Lipton RB. Predictors of treatment-response to caffeine combination products, acetaminophen, acetylsalicylic acid (aspirin), and nonsteroidal anti-inflammatory drugs in acute treatment of episodic migraine. *Headache*. 2023;63(3):342-352. doi:10.1111/head.14459
122. Schiano di Cola F, Bolchini M, Ceccardi G, et al. An observational study on monoclonal antibodies against calcitonin-gene-related peptide and its receptor. *Eur J Neurol*. 2023;30(6):1764-1773. doi:10.1111/ene.15761
123. Schiano di Cola F, Ceccardi G, Bolchini M, et al. Photophobia and migraine outcome during treatment with galcanezumab. *Front Neurol*. 2023;13:1088036. Published 2023 Jan 18. doi:10.3389/fneur.2022.1088036
124. Olgun, Aysu & Bayır, et al. The Relationship Between the Presence of Allodynia and Pain Acceptance and Somatosensory Amplification in Patients with Migraine. *Medical Bulletin of Haseki.* 2023 61. 113-119. 10.4274/haseki.galenos.2023.8817.
125. Hubig LT, Smith T, Williams E. et al*.* Measuring interictal burden among people affected by migraine: a descriptive survey study. *J Headache Pain* 23, 97 (2022). <https://doi.org/10.1186/s10194-022-01467-z>
126. Hrytsenko O, Kopchak O, Kozyk M, Strubchevska K. The impact of the COVID-19 pandemic on patients with migraine. *SAGE Open Medicine*. 2023;11. doi:10.1177/20503121231170726
127. De Icco R, Vaghi G, Allena M, et al. Does MIDAS reduction at 3 months predict the outcome of erenumab treatment? A real-world, open-label trial. *J Headache Pain*. 2022;23(1):123. Published 2022 Sep 17. doi:10.1186/s10194-022-01480-2
128. Asawavichienjinda T, Storer RJ. Preventive treatment response associated with migraine aura subtypes in a Thai population. *Front Hum Neurosci*. 2023;16:1065859. Published 2023 Jan 9. doi:10.3389/fnhum.2022.1065859
129. Abdelghaffar M, Hussein M, Thabet NH, et al*.* The potential impact of migraine headache on retinal nerve fiber layer thickness. *Egypt J Neurol Psychiatry Neurosurg* 58, 141 2022. <https://doi.org/10.1186/s41983-022-00570-x>
130. Liu Q, Liu F, Yu X, Zang J, Tan G. Telemedicine efficacy and satisfaction of patients and headache specialists in migraine management. *Front Mol Neurosci*. 2023;16:1093287. Published 2023 Mar 23. doi:10.3389/fnmol.2023.1093287
131. Kurtses Gürsoy B, Köseoğlu Toksoy C. Psychological Resilience and Stress Coping Styles in Migraine Patients. *Neuropsychiatr Dis Treat*. 2023;19:63-72. Published 2023 Jan 5. doi:10.2147/NDT.S398838
132. Andreou AP, Fuccaro M, Hill B et al*.* Two-year effectiveness of erenumab in resistant chronic migraine: a prospective real-world analysis. *J Headache Pain* 23, 139 2022. <https://doi.org/10.1186/s10194-022-01507-8>
133. Zhao H, Xiao Z, Zhang L, et al. Real-World Treatment Patterns and Outcomes Among Patients with Episodic Migraine in China: Results from the Adelphi Migraine Disease Specific Programme™. *J Pain Res*. 2023;16:357-371. Published 2023 Feb 3. doi:10.2147/JPR.S371887
134. Tsao YC, Wang YF, Fuh JL, et al. Non-aura visual disturbance with high visual aura rating scale scores has stronger association with migraine chronification than typical aura. *Cephalalgia*. 2022;42(14):1487-1497. doi:10.1177/03331024221123074
135. Onan D, Martelletti P. Does the Intensity of the Headache Differ According to the Level of Neck Disability in Chronic Migraine Patients?. *Int J Environ Res Public Health*. 2022;19(23):16307. Published 2022 Dec 6. doi:10.3390/ijerph192316307
136. MacGregor EA, Komori M, Krege JH, et al. Efficacy of lasmiditan for the acute treatment of perimenstrual migraine. *Cephalalgia*. 2022;42(14):1467-1475. doi:10.1177/03331024221118929
137. Varnado OJ, Ye W, Mi X, Burge R, Hall J. Annual indirect costs savings in patients with episodic or chronic migraine: a post-hoc analysis of phase 3 galcanezumab clinical trials in the United States. *J Med Econ*. 2023;26(1):149-157. doi:10.1080/13696998.2023.2165365
138. Arab A, Khorvash F, Karimi E, Hadi A, Askari G. Associations between adherence to Mediterranean dietary pattern and frequency, duration, and severity of migraine headache: A cross-sectional study. *Nutr Neurosci*. 2023;26(1):1-10. doi:10.1080/1028415X.2021.2009162
139. He N, Shao H, He J, Zhang X, Ye D, Lv Z. Evaluation of retinal vessel and perfusion density in migraine patients by optical coherence tomography angiography. *Photodiagnosis Photodyn Ther*. 2022;40:103060. doi:10.1016/j.pdpdt.2022.103060
140. Blumenfeld A, Ettrup A, Hirman J, Ebert B, Cady R. Long-term reductions in disease impact in patients with chronic migraine following preventive treatment with eptinezumab. *BMC Neurol*. 2022;22(1):251. Published 2022 Jul 8. doi:10.1186/s12883-022-02774-3
141. Gonzalez-Martinez A, Pagán J, Sanz-García A, et al. Machine-learning-based approach for predicting response to anti-calcitonin gene-related peptide (CGRP) receptor or ligand antibody treatment in patients with migraine: A multicenter Spanish study. *Eur J Neurol*. 2022;29(10):3102-3111. doi:10.1111/ene.15458
142. Rossi DM, Bevilaqua-Grossi D, Mascarenhas S. et al*.* Noninvasive intracranial pressure monitoring in women with migraine. *Sci Rep* 12, 2635 (2022). <https://doi.org/10.1038/s41598-022-06258-9>
143. Rosendale N, Guterman EL, Obedin-Maliver J, et al. Migraine, Migraine Disability, Trauma, and Discrimination in Sexual and Gender Minority Individuals [published online ahead of print, 2022 Jul 11]. *Neurology*. 2022;99(14):e1549-e1559. doi:10.1212/WNL.0000000000200941
144. Butt M, Chavarria Y, Ninmol J, et al. Association of increased pain intensity, daytime sleepiness, poor sleep quality, and quality of life with mobile phone overuse in patients with migraine: A multicenter, cross-sectional comparative study. *Brain Behav*. 2022;12(10):e2760. doi:10.1002/brb3.2760
145. Li Y, Chen G, Lv J, et al. Abnormalities in resting-state EEG microstates are a vulnerability marker of migraine. *J Headache Pain*. 2022;23(1):45. Published 2022 Apr 5. doi:10.1186/s10194-022-01414-y
146. Fofi L, Altamura C, Fiorentini G, et al. Improving distress perception and mutuality in migraine caregivers after 6 months of galcanezumab treatment. *Headache*. 2022;62(9):1143-1147. doi:10.1111/head.14400
147. Ray JC, Cheema S, Foster E, et al. Autonomic symptoms in migraine: Results of a prospective longitudinal study. *Front Neurol*. 2022;13:1036798. Published 2022 Nov 3. doi:10.3389/fneur.2022.1036798
148. Fernández-de-Las-Peñas C, Florencio LL, Varol U, Pareja JA, Ordás-Bandera C, Valera-Calero JA. Network Analysis Reveals That Headache-Related, Psychological and Psycho-Physical Outcomes Represent Different Aspects in Women with Migraine. *Diagnostics (Basel)*. 2022;12(10):2318. Published 2022 Sep 26. doi:10.3390/diagnostics12102318
149. Ghoreishy SM, Askari G, Mohammadi H, Campbell MS, Khorvash F, Arab A. Associations between potential inflammatory properties of the diet and frequency, duration, and severity of migraine headaches: a cross-sectional study. *Sci Rep*. 2022;12(1):2878. Published 2022 Feb 21. doi:10.1038/s41598-022-06819-y
150. Barbanti P, Egeo G, Aurilia C. et al*.* Fremanezumab in the prevention of high-frequency episodic and chronic migraine: a 12-week, multicenter, real-life, cohort study (the FRIEND study). *J Headache Pain* 23, 46 (2022). <https://doi.org/10.1186/s10194-022-01396-x>
151. Etefagh HH, Shahmiri SS, Melali H, et al. Bariatric Surgery in Migraine patients: CGRP Level and Weight Loss. *Obes Surg*. 2022;32(11):3635-3640. doi:10.1007/s11695-022-06218-2
152. Barbanti P, Egeo G, Aurilia C, et al. The first report of the Italian Migraine Registry (I-GRAINE). *Neurol Sci*. 2022;43(9):5725-5728. doi:10.1007/s10072-022-06214-5
153. Oh SY, Kang JJ, Kim S, Lee JM, Kim JS, Dieterich M. A preliminary trial of botulinum toxin type A in patients with vestibular migraine: A longitudinal fMRI study. *Front Neurol*. 2022;13:955158. Published 2022 Jul 25. doi:10.3389/fneur.2022.955158
154. Butt MN, Maryum M, Amjad I, Khan OJ, Awan L. Effects of aerobic exercise and progressive muscle relaxation on migraine. *J Pak Med Assoc*. 2022;72(6):1153-1157. doi:10.47391/JPMA.0838
155. Saeed H, Tulbah AS, Gamal A, Kamal M. Assessment and characteristics of Erenumab therapy on migraine management. *Saudi Pharm J*. 2022;30(8):1153-1158. doi:10.1016/j.jsps.2022.06.015
156. Wang YF, Liao YC, Tzeng YS, et al. Mutation screening and association analysis of *NOTCH3* p.R544C in patients with migraine with or without aura. *Cephalalgia*. 2022;42(9):888-898. doi:10.1177/03331024221080891
157. Morales Bacas E, Portilla Cuenca JC, Romero Cantero V et al. Experience with erenumab: Data from real clinical practice, *Neurology Perspectives* Volume 2, Issue 3, 2022 111-116 <https://doi.org/10.1016/j.neurop.2022.01.009>.
158. Arab A, Khorvash F, Heidari Z, Askari G. Is there a relationship between dietary sodium and potassium intake and clinical findings of a migraine headache?. *Br J Nutr*. 2022;127(12):1839-1848. doi:10.1017/S000711452100283X
159. Malik Y, et al. Paradigm shift in migraine management impacted by COVID-19 pandemic and the role of confounding factors inflicting the change. *Neurosciences Journal* 2022 27 (3) 156-163; DOI: 10.17712/nsj.2022.3.20210058
160. Cetta I, Messina R, Zanandrea L, Colombo B, Filippi M. Comparison of efficacy and safety of erenumab between over and under 65-year-old refractory migraine patients: a pivotal study. *Neurol Sci*. 2022;43(9):5769-5771. doi:10.1007/s10072-022-06190-w
161. Helli B, Anjirizadeh F, Mehramiri A, et al. The Effect of Ginger (Zingiber officinale Rosc.) Consumption in Headache Prophylaxis in Patients with Migraine: A Randomized Placebo-Controlled Clinical Trial*. Jundishapur J Nat Pharm Prod* 2022;17(3):e120449. <https://doi.org/10.5812/jjnpp-120449>.
162. Börner C, Renner T, Trepte-Freisleder F, et al. Response Predictors of Repetitive Neuromuscular Magnetic Stimulation in the Preventive Treatment of Episodic Migraine. *Front Neurol*. 2022;13:919623. Published 2022 Jul 28. doi:10.3389/fneur.2022.919623
163. Wang X, Zhang Y, Qi W, et al. Alteration in Functional Magnetic Resonance Imaging Signal Complexity Across Multiple Time Scales in Patients With Migraine Without Aura. *Front Neurosci*. 2022;16:825172. Published 2022 Mar 7. doi:10.3389/fnins.2022.825172
164. Tepper SJ, Ailani J, Ford JH, et al. Effects of Galcanezumab on Health-Related Quality of Life and Disability in Patients with Previous Failure of 2-4 Migraine Preventive Medication Categories: Results from a Phase IIIb Randomized, Placebo-Controlled, Multicenter Clinical Trial (CONQUER) [published correction appears in Clin Drug Investig. 2022 Mar 7;:]. *Clin Drug Investig*. 2022;42(3):263-275. doi:10.1007/s40261-021-01115-5
165. Dzator JSA, Howe PRC, Coupland KG, Wong RHX. A Randomised, Double-Blind, Placebo-Controlled Crossover Trial of Resveratrol Supplementation for Prophylaxis of Hormonal Migraine. *Nutrients*. 2022;14(9):1763. Published 2022 Apr 22. doi:10.3390/nu14091763
166. Chen Z, Zhao H, Chen X, et al. The increased iron deposition of the gray matter over the whole brain in chronic migraine: An exploratory quantitative susceptibility mapping study. *Mol Pain*. 2022;18:17448069221074987. doi:10.1177/17448069221074987
167. Vgontzas A, Mostofsky E, Hagan K, Rueschman M, Mittleman MA, Bertisch SM. Napping behavior in adults with episodic migraine: a six-week prospective cohort study. *Sleep*. 2022;45(3):zsab273. doi:10.1093/sleep/zsab273
168. Younis, et al. The Frequency of Fibromyalgia in Migraine Patients. Open Access Macedonian *Journal of Medical Sciences*. 2022 10. 260-264. 10.3889/oamjms.2022.8246.
169. Anushiravani M, Hosseini SM, Nikkhah K, et al. Evaluation of a Polyherbal Formulation on the Management of Migraine Headaches due to Functional Dyspepsia: A Double-Blind, Randomized, Placebo-Controlled Clinical Trial. *Evid Based Complement Alternat Med*. 2022;2022:9872933. Published 2022 Dec 3. doi:10.1155/2022/9872933
170. Shah J, et al. Repetitive transcranial magnetic stimulation for prophylactive treatment of chronic migraine: A randomised, single-blind, parallel-group, shamcontrolled trial. *Neurology Asia* 2022; 27(1): 137 – 144 <https://doi.org/10.54029/2022mau>
171. Klonowski T, Kropp P, Straube A, et al. Psychological factors associated with headache frequency, intensity, and headache-related disability in migraine patients. *Neurol Sci* 43, 1255–1266 2022. <https://doi.org/10.1007/s10072-021-05453-2>
172. Liu L, Lyu TL, Fu MY, et al. Changes in brain connectivity linked to multisensory processing of pain modulation in migraine with acupuncture treatment. *Neuroimage Clin*. 2022;36:103168. doi:10.1016/j.nicl.2022.103168
173. Hemasian H, Abedini F, Arab A, Khorvash F. A novel technique of botulinum toxin injection around skull sutures for chronic migraine: A randomized controlled clinical trial. *J Res Med Sci*. 2022;27:85. Published 2022 Nov 25. doi:10.4103/jrms.jrms_372_21
174. Suzuki K, Suzuki S, Shiina T, et al. Investigating the relationships between the burden of multiple sensory hypersensitivity symptoms and headache-related disability in patents with migraine. *J Headache Pain*. 2021;22(1):77. Published 2021 Jul 19. doi:10.1186/s10194-021-01294-8
175. Okonkwo R, Tockhorn-Heidenreich A, Stroud C, Paget MA, Matharu MS, Tassorelli C. Efficacy of galcanezumab in patients with migraine and history of failure to 3-4 preventive medication categories: subgroup analysis from CONQUER study. *J Headache Pain*. 2021;22(1):113. Published 2021 Sep 30. doi:10.1186/s10194-021-01322-7
176. Ornello R, Baraldi C, Guerzoni S, et al. Gender Differences in 3-Month Outcomes of Erenumab Treatment-Study on Efficacy and Safety of Treatment With Erenumab in Men. *Front Neurol*. 2021;12:774341. Published 2021 Dec 16. doi:10.3389/fneur.2021.774341
177. Ashina M, Cohen JM, Galic M, et al. Efficacy and safety of fremanezumab in patients with episodic and chronic migraine with documented inadequate response to 2 to 4 classes of migraine preventive medications over 6 months of treatment in the phase 3b FOCUS study. *J Headache Pain*. 2021;22(1):68. Published 2021 Jul 10. doi:10.1186/s10194-021-01279-7
178. Khani S, Hejazi SA, Yaghoubi M, Sharifipour E. Comparative study of magnesium, sodium valproate, and concurrent magnesium-sodium valproate therapy in the prevention of migraine headaches: a randomized controlled double-blind trial. *J Headache Pain*. 2021;22(1):21. Published 2021 Apr 7. doi:10.1186/s10194-021-01234-6
179. Jedynak J, Eross E, Gendolla A, Rettiganti M, Stauffer VL. Shift from high-frequency to low-frequency episodic migraine in patients treated with Galcanezumab: results from two global randomized clinical trials. *J Headache Pain*. 2021;22(1):48. Published 2021 May 28. doi:10.1186/s10194-021-01222-w
180. Parlongue G, Cerdan EV, Koenig J, Williams DP. Smartphone based music intervention in the treatment of episodic migraine headaches - A pilot trial. *Complement Ther Med*. 2021;63:102779. doi:10.1016/j.ctim.2021.102779
181. Vernieri F, Brunelli N, Messina R, et al. Discontinuing monoclonal antibodies targeting CGRP pathway after one-year treatment: an observational longitudinal cohort study. *J Headache Pain*. 2021;22(1):154. Published 2021 Dec 18. doi:10.1186/s10194-021-01363-y
182. Carvalho GF, Luedtke K, Braun T. Minimal important change and responsiveness of the Migraine Disability Assessment Score (MIDAS) questionnaire. *J Headache Pain*. 2021;22(1):126. Published 2021 Oct 21. doi:10.1186/s10194-021-01339-y
183. Kim BK, Chu MK, Yu SJ et al*.* Burden of migraine and unmet needs from the patients’ perspective: a survey across 11 specialized headache clinics in Korea. *J Headache Pain* 22, 45 2021. <https://doi.org/10.1186/s10194-021-01250-6>
184. Peek AL, Leaver AM, Foster S, et al. Increase in ACC GABA+ levels correlate with decrease in migraine frequency, intensity and disability over time. *J Headache Pain*. 2021;22(1):150. Published 2021 Dec 13. doi:10.1186/s10194-021-01352-1
185. Blumenfeld AM, Frishberg BM, Schim JD, et al. Real-World Evidence for Control of Chronic Migraine Patients Receiving CGRP Monoclonal Antibody Therapy Added to OnabotulinumtoxinA: A Retrospective Chart Review. *Pain Ther*. 2021;10(2):809-826. doi:10.1007/s40122-021-00264-x
186. Belvís R, Irimia P, Pozo-Rosich P. et al*.* MAB-MIG: registry of the spanish neurological society of erenumab for migraine prevention. *J Headache Pain* 22, 74 (2021). <https://doi.org/10.1186/s10194-021-01267-x>
187. Vernieri F, Altamura C, Brunelli N. et al*.* Galcanezumab for the prevention of high frequency episodic and chronic migraine in real life in Italy: a multicenter prospective cohort study (the GARLIT study). *J Headache Pain* 22, 35 (2021). <https://doi.org/10.1186/s10194-021-01247-1>
188. Ferreira PL, Luzeiro I, Lopes M, Jorge A, Silva B, Ferreira L. Validity and reliability of the Portuguese version of the modified Migraine Disability Assessment. *BMC Neurol*. 2021;21(1):58. Published 2021 Feb 6. doi:10.1186/s12883-021-02085-z
189. Silberstein SD, Cohen JM, Yang R. et al*.* Treatment benefit among migraine patients taking fremanezumab: results from a post hoc responder analysis of two placebo-controlled trials. *J Headache Pain* 22, 2 (2021). <https://doi.org/10.1186/s10194-020-01212-4>
190. Domingues FS, Gayoso MV, Sikandar S, da Silva LM, Fonseca RG, de Barros GAM. Analgesic efficacy of a portable, disposable, and self-applied transcutaneous electrical nerve stimulation device during migraine attacks: A real-life randomized controlled trial. *Pain Pract*. 2021;21(8):850-858. doi:10.1111/papr.13042
191. Hammad AB, Elsharkawy RE, et al. Repetitive transcranial magnetic stimulation as a prophylactic treatment in migraine. *Egypt J Neurol Psychiatry Neurosurg* 57, 5 2021. <https://doi.org/10.1186/s41983-020-00254-4>
192. Torres-Ferrús M, Gallardo VJ, Alpuente A, Caronna E, Gine-Cipres E, Pozo-Rosich P. The impact of anti-CGRP monoclonal antibodies in resistant migraine patients: a real-world evidence observational study. *J Neurol*. 2021;268(10):3789-3798. doi:10.1007/s00415-021-10523-8
193. Luo JM, Liu EZ, Yang HD, et al. Prevalence and Factors Associated With Suicidal Ideation in Medical Students With Migraine. *Front Psychiatry*. 2021;12:683342. Published 2021 Oct 21. doi:10.3389/fpsyt.2021.683342
194. De Luca C, Baldacci F, Mazzucchi S, et al. CGRP Inhibitors and Oxidative Stress Biomarkers in Resistant Migraine: A Real-Life Study with Erenumab, Fremanezumab, and Galcanezumab. *J Clin Med*. 2021;10(19):4586. Published 2021 Oct 4. doi:10.3390/jcm10194586
195. Wang SJ, Roxas AA Jr, Saravia B, et al. Randomised, controlled trial of erenumab for the prevention of episodic migraine in patients from Asia, the Middle East, and Latin America: The EMPOwER study. *Cephalalgia*. 2021;41(13):1285-1297. doi:10.1177/03331024211024160
196. Gil-Gouveia R. "Headache yesterday" at work. Pilot study of headache impact in an active workforce. *Rev Neurol (Paris)*. 2021;177(9):1189-1194. doi:10.1016/j.neurol.2020.12.013
197. Memmedova F, Emre U, Yalın OÖ, Doğan OC. Evaluation of temporomandibular joint disorder in headache patients. *Neurol Sci*. 2021;42(11):4503-4509. doi:10.1007/s10072-021-05119-z
198. Kim S, Bae DW, Park SG, Park JW. The impact of Pain-related emotions on migraine. *Sci Rep*. 2021;11(1):577. Published 2021 Jan 12. doi:10.1038/s41598-020-80094-7
199. Maraia Z, Ricci D, Rocchi MBL, et al. Real-Life Analysis with Erenumab: First Target Therapy in the Episodic and Chronic Migraine's Prophylaxis. *J Clin Med*. 2021;10(19):4425. Published 2021 Sep 27. doi:10.3390/jcm10194425
200. Grazzi L, Grignani E, Raggi A, Rizzoli P, Guastafierro E. Effect of a Mindfulness-Based Intervention for Chronic Migraine and High Frequency Episodic Migraine in Adolescents: A Pilot Single-Arm Open-Label Study. *Int J Environ Res Public Health*. 2021;18(22):11739. Published 2021 Nov 9. doi:10.3390/ijerph182211739
201. Ishii R, Schwedt TJ, Dumkrieger G, et al. Chronic versus episodic migraine: The 15-day threshold does not adequately reflect substantial differences in disability across the full spectrum of headache frequency. *Headache*. 2021;61(7):992-1003. doi:10.1111/head.14154
202. Hirata K, Ueda K, Komori M, et al. Unmet Needs in Japanese Patients Who Report Insufficient Efficacy with Triptans for Acute Treatment of Migraine: Retrospective Analysis of Real-World Data. *Pain Ther*. 2021;10(1):415-432. doi:10.1007/s40122-020-00223-y
203. Pravatà E, Riccitelli GC, Sestieri C, et al. Migraine in Multiple Sclerosis Patients Affects Functional Connectivity of the Brain Circuitry Involved in Pain Processing. *Front Neurol*. 2021;12:690300. Published 2021 Aug 12. doi:10.3389/fneur.2021.690300
204. Muñoz-Gómez E, Inglés M, Serra-Añó P, Espí-López GV. Effectiveness of a manual therapy protocol based on articulatory techniques in migraine patients. A randomized controlled trial. *Musculoskelet Sci Pract*. 2021;54:102386. doi:10.1016/j.msksp.2021.102386
205. Grazzi L, Andrasik F, Rizzoli P, Bernstein C, Sansone E, Raggi A. Acceptance and commitment therapy for high frequency episodic migraine without aura: Findings from a randomized pilot investigation. *Headache*. 2021;61(6):895-905. doi:10.1111/head.14139
206. Estave PM, Beeghly S, Anderson R, et al. Learning the full impact of migraine through patient voices: A qualitative study. *Headache*. 2021;61(7):1004-1020. doi:10.1111/head.14151
207. Sakai F, Takeshima T, Homma G, Tanji Y, Katagiri H, Komori M. Phase 2 randomized placebo-controlled study of lasmiditan for the acute treatment of migraine in Japanese patients. *Headache*. 2021;61(5):755-765. doi:10.1111/head.14122
208. Short AL. Enhancing migraine self-efficacy and reducing disability through a self-management program. *J Am Assoc Nurse Pract*. 2019;33(1):20-28. Published 2019 Nov 13. doi:10.1097/JXX.0000000000000323
209. Wells RE, O'Connell N, Pierce CR, et al. Effectiveness of Mindfulness Meditation vs Headache Education for Adults With Migraine: A Randomized Clinical Trial. *JAMA Intern Med*. 2021;181(3):317-328. doi:10.1001/jamainternmed.2020.7090
210. Hagan KK, Li W, Mostofsky E, et al. Prospective cohort study of routine exercise and headache outcomes among adults with episodic migraine. *Headache*. 2021;61(3):493-499. doi:10.1111/head.14037
211. Li YF, Hu HM, Wang BN, et al. Efficacy and Safety of Chuanxiong Qingnao Granule in Patients with Migraine: A Randomized, Double-Blind, Placebo-Controlled Trial. *Evid Based Complement Alternat Med*. 2021;2021:6203999. Published 2021 Dec 22. doi:10.1155/2021/6203999
212. Silvestro M, Tessitore A, Scotto di Clemente F, Battista G, Tedeschi G, Russo A. Additive Interaction Between Onabotulinumtoxin-A and Erenumab in Patients With Refractory Migraine. *Front Neurol*. 2021;12:656294. Published 2021 Apr 8. doi:10.3389/fneur.2021.656294
213. Minen MT, Friedman BW, Adhikari S, et al. Introduction of a smartphone based behavioral intervention for migraine in the emergency department. *Gen Hosp Psychiatry*. 2021;69:12-19. doi:10.1016/j.genhosppsych.2020.12.009
214. Ashina M, Reuter U, Smith T, et al. Randomized, controlled trial of lasmiditan over four migraine attacks: Findings from the CENTURION study. *Cephalalgia*. 2021;41(3):294-304. doi:10.1177/0333102421989232
215. Sakai F, Takeshima T, Tatsuoka Y, et al. Long-term efficacy and safety during open-label erenumab treatment in Japanese patients with episodic migraine. *Headache*. 2021;61(4):653-661. doi:10.1111/head.14096
216. Sharawat IK, Panda PK. Caregiver Satisfaction and Effectiveness of Teleconsultation in Children and Adolescents With Migraine During the Ongoing COVID-19 Pandemic. *J Child Neurol*. 2021;36(4):296-303. doi:10.1177/0883073820968653
217. Alkhaffaf WH, Almahdawi AM Efficacy and tolerability of melatonin versus topiramate in migraine prevention. *Archivos Venezolanos de Farmacologia y Terapeutica*, 2021 40 (1), pp. 27-32. DOI: 10.5281/zenodo.4660433
218. Mehta JN, Parikh S, Desai SD, Solanki RC, G Pathak A. Study of Additive Effect of Yoga and Physical Therapies to Standard Pharmacologic Treatment in Migraine. *J Neurosci Rural Pract*. 2021;12(1):60-66. doi:10.1055/s-0040-1718842
219. Parohan M, Sarraf P, Javanbakht MH, Foroushani AR, Ranji-Burachaloo S, Djalali M. The synergistic effects of nano-curcumin and coenzyme Q10 supplementation in migraine prophylaxis: a randomized, placebo-controlled, double-blind trial. *Nutr Neurosci*. 2021;24(4):317-326. doi:10.1080/1028415X.2019.1627770
220. Amini L, Yaghini O, Ghazavi M, Aslani N. L-carnitine versus Propranolol for pediatric migraine prophylaxis. *Iran J Child Neurol*. 2021;15(2):77-86. doi:10.22037/ijcn.v15i2.25558
221. Tehrani M, Ghoreishi A, et al. Biofeedback: An Effective Add-on Treatment for Migraine Headache Alongside Medication Therapy. *Journal of Advances in Medical and Biomedical Research*. 2021 29. 14-20. 10.30699/jambs.29.132.14.
222. Rua T, Mazumder A, Akande Y, et al. Management of chronic headache with referral from primary care to direct access to MRI compared with Neurology services: an observational prospective study in London. *BMJ Open*. 2020;10(10):e036097. Published 2020 Oct 16. doi:10.1136/bmjopen-2019-036097
223. Ford JH, Kurth T, Starling AJ, et al. Migraine Headache Day Response Rates and the Implications to Patient Functioning: An Evaluation of 3 Randomized Phase 3 Clinical Trials of Galcanezumab in Patients With Migraine [published correction appears in Headache. 2021 Jun;61(6):977]. *Headache*. 2020;60(10):2304-2319. doi:10.1111/head.14013
224. Hamamci M, Karasalan Ö, İnan LE. Can personality traits, obesity, depression, anxiety, and quality of life explain the association between migraine and disordered eating attitudes?. *Arq Neuropsiquiatr*. 2020;78(9):541-548. doi:10.1590/0004-282x20200046
225. Fallah R, Sarraf Yazd S, Sohrevardi SM. Efficacy of Topiramate Alone and Topiramate Plus Vitamin D3 in the Prophylaxis of Pediatric Migraine: A Randomized Clinical Trial. *Iranian Journal of Child Neurology*, 2020 *14*(4), 77–86. <https://doi.org/10.22037/ijcn.v15i1.18017>
226. Wei HL, Chen J, Chen YC, et al. Impaired effective functional connectivity of the sensorimotor network in interictal episodic migraineurs without aura. *J Headache Pain*. 2020;21(1):111. Published 2020 Sep 14. doi:10.1186/s10194-020-01176-5
227. Woldeamanuel YW, Sanjanwala BM, Peretz AM, Cowan RP. Exploring Natural Clusters of Chronic Migraine Phenotypes: A Cross-Sectional Clinical Study. *Sci Rep*. 2020;10(1):2804. Published 2020 Feb 18. doi:10.1038/s41598-020-59738-1
228. Amin R, Emara T, Ashour S. et al*.* The role of left prefrontal transcranial magnetic stimulation in episodic migraine prophylaxis. *Egypt J Neurol Psychiatry Neurosurg* 56, 19 2020. <https://doi.org/10.1186/s41983-019-0140-5>
229. Cheng S, Jenkins B, Limberg N, Hutton E. Erenumab in Chronic Migraine: An Australian Experience. *Headache*. 2020;60(10):2555-2562. doi:10.1111/head.13968
230. Nemichandra SC, Pradeep R, Harsha S, Radhika K, Iqbal R. Erectile Dysfunction in Migraine in Indian Patients. *Ann Indian Acad Neurol*. 2020;23(6):792-795. doi:10.4103/aian.AIAN_554_19
231. Torres-Ferrus M, Gallardo VJ, Alpuente A, Pozo-Rosich P. Influence of headache pain intensity and frequency on migraine-related disability in chronic migraine patients treated with OnabotulinumtoxinA. *J Headache Pain*. 2020;21(1):88. Published 2020 Jul 11. doi:10.1186/s10194-020-01157-8
232. Russo A, Silvestro M, Scotto di Clemente F, et al. Multidimensional assessment of the effects of erenumab in chronic migraine patients with previous unsuccessful preventive treatments: a comprehensive real-world experience. *J Headache Pain*. 2020;21(1):69. Published 2020 Jun 9. doi:10.1186/s10194-020-01143-0
233. Khorsha F, Mirzababaei A, Togha M, Mirzaei K. Association of drinking water and migraine headache severity. *J Clin Neurosci*. 2020;77:81-84. doi:10.1016/j.jocn.2020.05.034
234. Vikelis M, Dermitzakis EV, Vlachos GS, et al. Open Label Prospective Experience of Supplementation with a Fixed Combination of Magnesium, Vitamin B2, Feverfew, Andrographis Paniculata and Coenzyme Q10 for Episodic Migraine Prophylaxis. *J Clin Med*. 2020;10(1):67. Published 2020 Dec 27. doi:10.3390/jcm10010067
235. Dindo LN, Recober A, Calarge CA, et al. One-Day Acceptance and Commitment Therapy Compared to Support for Depressed Migraine Patients: a Randomized Clinical Trial. *Neurotherapeutics*. 2020;17(2):743-753. doi:10.1007/s13311-019-00818-0
236. Kumar A, Bhatia R, Sharma G, et al. Effect of yoga as add-on therapy in migraine (CONTAIN): A randomized clinical trial. *Neurology*. 2020;94(21):e2203-e2212. doi:10.1212/WNL.0000000000009473
237. Fernandes L, Khan N, Dobson J, Randall M, Idrovo L. Multiple Cranial Nerve Blocks as an Alternative Preventative Therapy for Chronic Migraine. *Headache*. 2020;60(5):981-987. doi:10.1111/head.13792
238. Ghorbani Z, Rafiee P, Fotouhi A, et al. The effects of vitamin D supplementation on interictal serum levels of calcitonin gene-related peptide (CGRP) in episodic migraine patients: post hoc analysis of a randomized double-blind placebo-controlled trial. *J Headache Pain*. 2020;21(1):22. Published 2020 Feb 24. doi:10.1186/s10194-020-01090-w
239. Lombard L, Farrar M, Ye W, et al. A global real-world assessment of the impact on health-related quality of life and work productivity of migraine in patients with insufficient versus good response to triptan medication. *J Headache Pain*. 2020;21(1):41. Published 2020 Apr 29. doi:10.1186/s10194-020-01110-9
240. Sakai F, Takeshima T, Tatsuoka Y, et al. A Randomized Phase 2 Study of Erenumab for the Prevention of Episodic Migraine in Japanese Adults. *Headache*. 2019;59(10):1731-1742. doi:10.1111/head.13652
241. Alpuente A, Gallardo VJ, Torres-Ferrus M, Alvarez-Sabin J, Pozo-Rosich P. Early efficacy and late gain in chronic and high-frequency episodic migraine with onabotulinumtoxinA. *Eur J Neurol*. 2019;26(12):1464-1470. doi:10.1111/ene.14028
242. Minen M, Adhikari S, Seng E, et al. Smartphone-based migraine behavioral therapy: a single-arm study with assessment of mental health predictors. 2019 *npj Digital Medicine* 2:46 ; <https://doi.org/10.1038/s41746-019-0116-y>
243. Yu S, Ran Y, Wan Q, et al. Efficacy and Safety of Toutongning Capsule in Patients with Migraine: A Multicenter, Randomized, Double-Blind, Placebo-Controlled Trial. *J Altern Complement Med*. 2019;25(12):1215-1224. doi:10.1089/acm.2018.0500
244. Akarsu EO, Baykan B, Ertaş M, et al. Sex Differences of Migraine: Results of a Nationwide Home-based Study in Turkey. *Noro Psikiyatr Ars*. 2019;57(2):126-130. Published 2019 Sep 26. doi:10.29399/npa.23240
245. Fernández-de-Las-Peñas C, Ambite-Quesada S, Florencio LL, Palacios-Ceña M, Ordás-Bandera C, Arendt-Nielsen L. Catechol-O-Methyltransferase Val158Met Polymorphism Is Associated with Anxiety, Depression, and Widespread Pressure Pain Sensitivity in Women with Chronic, but Not Episodic, Migraine. *Pain Med*. 2019;20(7):1409-1417. doi:10.1093/pm/pny237
246. Pradeep R, Sundarmurthy H, Karan V, Kulkarni P. Prevalence and Predictors of Female Sexual Dysfunction in Migraine. *Ann Indian Acad Neurol*. 2019;22(3):291-294. doi:10.4103/aian.AIAN_508_18
247. Kim B, Kyung Chu M, et al. Long-term safety and efficacy of onabotulinumtoxinA for the prevention of chronic migraine in a South Korean population: COMPEL study *Neurology Asia* 2019; 24(2) : 127 – 137
248. Forcelini CM, Ramos M, Santos IFD, et al. The influence of allergic rhinoconjunctivitis on migraine disability in children. *Arq Neuropsiquiatr*. 2019;77(6):418-423. Published 2019 Jul 15. doi:10.1590/0004-282X20190058
249. Doty EG, Krege JH, Jin L, Raskin J, Halker Singh RB, Kalidas K. Sustained responses to lasmiditan: Results from post-hoc analyses of two Phase 3 randomized clinical trials for acute treatment of migraine. *Cephalalgia*. 2019;39(12):1569-1576. doi:10.1177/0333102419859313
250. Stark C, Stark R, Limberg N, et al. Real-world effectiveness of onabotulinumtoxinA treatment for the prevention of headaches in adults with chronic migraine in Australia: a retrospective study. *J Headache Pain*. 2019;20(1):81. Published 2019 Jul 15. doi:10.1186/s10194-019-1030-z
251. Forcelini CM, Gradaschi RTS, Tonin GA, et al. Is allergic rhinitis related to migraine disability in adults?. *Arq Neuropsiquiatr*. 2019;77(6):424-428. Published 2019 Jul 15. doi:10.1590/0004-282X20190063
252. Ruscheweyh R, Pereira D, Hasenbring MI, Straube A. Pain-related avoidance and endurance behaviour in migraine: an observational study. *J Headache Pain*. 2019;20(1):9. Published 2019 Jan 18. doi:10.1186/s10194-019-0962-7
253. Sahu AK, Sinha VK, Goyal N. Effect of adjunctive intermittent theta-burst repetitive transcranial magnetic stimulation as a prophylactic treatment in migraine patients: A double-blind sham-controlled study. *Indian J Psychiatry*. 2019;61(2):139-145. doi:10.4103/psychiatry.IndianJPsychiatry_472_18
254. Gazerani P, Fuglsang R, Pedersen JG, et al. A randomized, double-blinded, placebo-controlled, parallel trial of vitamin D_3_ supplementation in adult patients with migraine. *Curr Med Res Opin*. 2019;35(4):715-723. doi:10.1080/03007995.2018.1519503
255. de Tommaso M, Brighina F, Delussi M. Effects of Botulinum Toxin A on Allodynia in Chronic Migraine: An Observational Open-Label Two-Year Study. *Eur Neurol*. 2019;81(1-2):37-46. doi:10.1159/000499764
256. Tepper SJ, Diener HC, Ashina M, et al. Erenumab in chronic migraine with medication overuse: Subgroup analysis of a randomized trial. *Neurology*. 2019;92(20):e2309-e2320. doi:10.1212/WNL.0000000000007497
257. Song TJ, Cho SJ, Kim WJ, Yang KI, Yun CH, Chu MK. Sex Differences in Prevalence, Symptoms, Impact, and Psychiatric Comorbidities in Migraine and Probable Migraine: A Population-Based Study. *Headache*. 2019;59(2):215-223. doi:10.1111/head.13470
258. Schiano di Cola F, Caratozzolo S, Liberini P, Rao R, Padovani A. Response Predictors in Chronic Migraine: Medication Overuse and Depressive Symptoms Negatively Impact Onabotulinumtoxin-A Treatment. *Front Neurol*. 2019;10:678. Published 2019 Jul 10. doi:10.3389/fneur.2019.00678
259. Shahnawaz K, Mughal BB, Madni B, et al. Comparison of efficacy and tolerability of melatonin and amitriptyline in children suffering with migraine. *Medical forum monthly* 2019 30(5), 12‐15
260. Bottiroli S, Galli F, Viana M, et al. Negative Short-Term Outcome of Detoxification Therapy in Chronic Migraine With Medication Overuse Headache: Role for Early Life Traumatic Experiences and Recent Stressful Events. *Front Neurol*. 2019;10:173. Published 2019 Mar 7. doi:10.3389/fneur.2019.00173
261. Camporeale A, Kudrow D, Sides R, et al. A phase 3, long-term, open-label safety study of Galcanezumab in patients with migraine. *BMC Neurol*. 2018;18(1):188. Published 2018 Nov 9. doi:10.1186/s12883-018-1193-2
262. Hussein M, Fathy W, Abd Elkareem RM. The potential role of serum vitamin D level in migraine headache: a case-control study. *J Pain Res*. 2019;12:2529-2536. Published 2019 Aug 20. doi:10.2147/JPR.S216314
263. Starikova NL, Baidina TV, Kalashnikova TP. Thyrotropin levels and severity of symptoms in migraine patients of tertiary headache center. *Cephalalgia*. 2019;39(1):148-152. doi:10.1177/0333102418794941
264. Mose LS, Pedersen SS, Debrabant B, Jensen RH, Gram B. The role of personality, disability and physical activity in the development of medication-overuse headache: a prospective observational study. *J Headache Pain*. 2018;19(1):39. Published 2018 May 25. doi:10.1186/s10194-018-0863-1
265. Ghazavi M, Jelodar G, Yaghini O, et al. The efficacy and safety of levetiracetam in the prophylaxis of migraine headaches in children – a randomised trial 2019 *Pediatria Polska* DOI:[10.5114/polp.2019.86434](http://dx.doi.org/10.5114/polp.2019.86434)
266. Jung Y, Won B, Lee M, Chung J, Han SJ, Kim M. The Efficacy of Shinbaro for the Preventive Treatment of Migraine: A Pilot Study. *Evid Based Complement Alternat Med*. 2019;2019:2363420. Published 2019 May 13. doi:10.1155/2019/2363420
267. Russo A, Santangelo G, Tessitore A, et al. Coping Strategies in Migraine without Aura: A Cross-Sectional Study. *Behav Neurol*. 2019;2019:5808610. Published 2019 May 5. doi:10.1155/2019/5808610
268. Yu Z, Wang R, Ao R, Yu S. Neck pain in episodic migraine: a cross-sectional study. *J Pain Res*. 2019;12:1605-1613. Published 2019 May 20. doi:10.2147/JPR.S200606
269. Cotta Ramusino M, De Cillis I, Costa A, Antonaci F. Impact of Medical Care on Symptomatic Drug Consumption and Quality of Life in Headache: A One-Year Population Study. *Front Neurol*. 2019;10:629. Published 2019 Jun 18. doi:10.3389/fneur.2019.00629
270. Velasco-Juanes F, Gómez-Esteban JC, et al. Clinical treatment of chronic and episodic migraine with onabotulinumtoxinA in a real-world setting. *Drugs & Therapy Perspectives*. 2018 34. 10.1007/s40267-018-0511-5.
271. Thakur E, Recober A, Turvey C, Dindo LN. Benefits of an on-line migraine education video for patients with co-occurring migraine and depression. *J Psychosom Res*. 2018;112:47-52. doi:10.1016/j.jpsychores.2018.06.012
272. Seng EK, Kuka AJ, Mayson SJ, Smitherman TA, Buse DC. Acceptance, Psychiatric Symptoms, and Migraine Disability: An Observational Study in a Headache Center. *Headache*. 2018;58(6):859-872. doi:10.1111/head.13325
273. Vetvik KG, MacGregor EA, Lundqvist C, Russell MB. Symptoms of premenstrual syndrome in female migraineurs with and without menstrual migraine. *J Headache Pain*. 2018;19(1):97. Published 2018 Oct 17. doi:10.1186/s10194-018-0931-6
274. Rościszewska-Żukowska I, Zając-Mnich M, Janik P. Characteristics and clinical correlates of white matter changes in brain magnetic resonance of migraine females. *Neurol Neurochir Pol*. 2018;52(6):695-703. doi:10.1016/j.pjnns.2018.09.007
275. Caronna E, Gallardo VJ, Hernández-Beltrán N, et al. OnabotulinumtoxinA: An Effective Tool in the Therapeutic Arsenal for Chronic Migraine With Medication Overuse. *Frontiers in neurology*. 2018 *9*, 808. <https://doi.org/10.3389/fneur.2018.00808>
276. Ojha P, Malhotra V, Pandey N. Association between Generalized Obesity and Migraine Features in Indian Females. *Indian Journalof Physiol Pharmacol* 2018 62(4) :453 -457.
277. Fallah R, Fazeli Shoroki F, Sekhavat L. A randomised clinical trial of efficacy of melatonin and amitriptyline in migraine prophylaxis of children. Iranian Journal of Child Neurology 2018. 12(1), 47–54. <https://doi.org/10.22037/ijcn.v12i1.12004>
278. Song TJ, Yun CH, Cho SJ, Kim WJ, Yang KI, Chu MK. Short sleep duration and poor sleep quality among migraineurs: A population-based study. *Cephalalgia*. 2018;38(5):855-864. doi:10.1177/0333102417716936
279. Ford JH, Foster SA, Stauffer VL, et al. Patient satisfaction, health care resource utilization, and acute headache medication use with galcanezumab: results from a 12-month open-label study in patients with migraine. *Patient preference and adherence*, 2018. *12*, 2413–2424. <https://doi.org/10.2147/PPA.S182563>
280. Zeybek S, Kisabay A, Sari U, et al. Evaluation of the effect of botulinum neurotoxin type A (BoNT/A) on daily activity performance in chronic migraine patients using VAS, MIDAS AND HIT-6 tests. Neurology Asia 2018; 23(1) : 35 – 43
281. Dahri M, Hashemilar M, et al. Efficacy of coenzyme Q10 for the prevention of migraine in women: A randomized, double-blind, placebo-controlled study. Eur Jour of Integrative Medicine 2017 Vol.16 8-14, ISSN 1876-3820, <https://doi.org/10.1016/j.eujim.2017.10.003>.
282. Matharu M, Halker R, Pozo-Rosich P, DeGryse R, Manack Adams A, Aurora SK. The impact of onabotulinumtoxinA on severe headache days: PREEMPT 56-week pooled analysis. *J Headache Pain*. 2017;18(1):78. doi:10.1186/s10194-017-0784-4
283. Ashina M, Dodick D, Goadsby PJ, et al. Erenumab (AMG 334) in episodic migraine: Interim analysis of an ongoing open-label study. *Neurology*. 2017;89(12):1237-1243. doi:10.1212/WNL.0000000000004391
284. Sarchielli P, Romoli M, Corbelli I, et al. Stopping Onabotulinum Treatment after the First Two Cycles Might Not Be Justified: Results of a Real-life Monocentric Prospective Study in Chronic Migraine. *Front Neurol*. 2017;8:655. Published 2017 Dec 4. doi:10.3389/fneur.2017.00655
285. Sampaio Rocha-Filho PA, Hershey AD. Pediatric Migraine Disability Assessment (PedMIDAS): Translation Into Brazilian Portuguese and Cross-Cultural Adaptation. *Headache*. 2017;57(9):1409-1415. doi:10.1111/head.13159
286. Grazzi L, Sansone E, Raggi A, et al. Mindfulness and pharmacological prophylaxis after withdrawal from medication overuse in patients with Chronic Migraine: an effectiveness trial with a one-year follow-up. *J Headache Pain*. 2017;18(1):15. doi:10.1186/s10194-017-0728-z
287. Mekhail NA, Estemalik E, Azer G, Davis K, Tepper SJ. Safety and Efficacy of Occipital Nerves Stimulation for the Treatment of Chronic Migraines: Randomized, Double-blind, Controlled Single-center Experience. *Pain Pract*. 2017;17(5):669-677. doi:10.1111/papr.12504
288. Powers S, Coffey S, Chamberlin L, et al. Trial of Amitriptyline, Topiramate, and Placebo for Pediatric Migraine. *N Engl J Med* 2017; 376:115-124 DOI: 10.1056/NEJMoa1610384
289. Bektas H, Karabulut H, Doganay B, Acar B. Allergens might trigger migraine attacks. *Acta Neurol Belg*. 2017;117(1):91-95. doi:10.1007/s13760-016-0645-y
290. Raggi A, Grazzi L, Ayadi R, et al. Clinical and psychosocial features of frequent relapsers (FR) among patients with chronic migraine and medication overuse. *Neurol Sci*. 2017;38(Suppl 1):169-171. doi:10.1007/s10072-017-2894-9
291. Shehata HS, Esmail EH, Abdelalim A, et al. Repetitive transcranial magnetic stimulation versus botulinum toxin injection in chronic migraine prophylaxis: a pilot randomized trial. *J Pain Res*. 2016;9:771-777. Published 2016 Oct 7. doi:10.2147/JPR.S116671
292. Kim J, Cho SJ, Kim WJ, Yang KI, Yun CH, Chu MK. Insomnia in probable migraine: a population-based study. *J Headache Pain*. 2016;17(1):92. doi:10.1186/s10194-016-0681-2
293. Lee MJ, Choi HA, Choi H, Chung CS. Caffeine discontinuation improves acute migraine treatment: a prospective clinic-based study. *J Headache Pain*. 2016;17(1):71. doi:10.1186/s10194-016-0662-5
294. Raggi A, Covelli V, Schiavolin S, et al. Psychosocial difficulties in patients with episodic migraine: a cross-sectional study. *Neurol Sci*. 2016;37(12):1979-1986. doi:10.1007/s10072-016-2705-8
295. Fischer M, Frank F, Wille G, et al. Triptans for Acute Migraine Headache: Current Experience With Triptan Use and Prescription Habits in a Tertiary Care Headache Outpatient Clinic: An Observational Study. *Headache* 2016 <https://doi.org/10.1111/head.12820>
296. Lucchesi C, Baldacci F, Cafalli M, et al. Fatigue, sleep-wake pattern, depressive and anxiety symptoms and body-mass index: analysis in a sample of episodic and chronic migraine patients. *Neurol Sci*. 2016;37(6):987-989. doi:10.1007/s10072-016-2505-1
297. Kim SY, Park SP. Cutaneous Allodynia and Its Risk Factors in Korean Patients with Migraine: A Survey of Two Tertiary Care Hospitals. *J Oral Facial Pain Headache*. 2016;30(4):323-329. doi:10.11607/ofph.1687
298. Gil-Gouveia R, Oliveira AG, Martins IP. The impact of cognitive symptoms on migraine attack-related disability. *Cephalalgia*. 2016;36(5):422-430. doi:10.1177/0333102415604471
299. Kollewe K, Escher CM, Wulff DU, et al. Long-term treatment of chronic migraine with OnabotulinumtoxinA: efficacy, quality of life and tolerability in a real-life setting. *J Neural Transm (Vienna)*. 2016;123(5):533-540. doi:10.1007/s00702-016-1539-0
300. Rafie S, Namjoyan F, Golfakhrabadi F,et al. Effect of lavender essential oil as a prophylactic therapy for migraine: A randomized controlled clinical trial. *Journal of Herbal Medicine* 2016, 18-23, ISSN 2210-8033, <https://doi.org/10.1016/j.hermed.2016.01.003>.
301. Berra E, Sances G, De Icco R, et al. Cost of Chronic and Episodic Migraine. A pilot study from a tertiary headache centre in northern Italy. *J Headache Pain*. 2015;16:532. doi:10.1186/s10194-015-0532-6
302. Demirci K, Demirci S, Akpinar A, Demirdaş A, Atay İM. Evaluation of Eating Attitude in Patients with Migraine. *Noro Psikiyatr Ars*. 2015;52(4):367-370. doi:10.5152/npa.2015.9997
303. Seo JG, Park SP. Validation of the Generalized Anxiety Disorder-7 (GAD-7) and GAD-2 in patients with migraine. *J Headache Pain*. 2015;16:97. doi:10.1186/s10194-015-0583-8
304. Liu HY, Fuh JL, Lin YY, Chen WT, Wang SJ. Suicide risk in patients with migraine and comorbid fibromyalgia. *Neurology*. 2015;85(12):1017-1023. doi:10.1212/WNL.0000000000001943
305. Bond DS, Buse DC, Lipton RB, et al. Clinical Pain Catastrophizing in Women With Migraine and Obesity. *Headache*. 2015;55(7):923-933. doi:10.1111/head.12597
306. Gómez-Beldarrain M, Anton-Ladislao A, Aguirre-Larracoechea U, Oroz I, García-Moncó JC. Low cognitive reserve is associated with chronic migraine with medication overuse and poor quality of life. *Cephalalgia*. 2015;35(8):683-691. doi:10.1177/0333102414553822
307. Silberstein SD, Dodick DW, Aurora SK, et al. Per cent of patients with chronic migraine who responded per onabotulinumtoxinA treatment cycle: PREEMPT. *J Neurol Neurosurg Psychiatry*. 2015;86(9):996-1001. doi:10.1136/jnnp-2013-307149
308. Seo JG, Park SP. Validation of the Patient Health Questionnaire-9 (PHQ-9) and PHQ-2 in patients with migraine. *J Headache Pain*. 2015;16:65. doi:10.1186/s10194-015-0552-2
309. Negro A, Curto M, Lionetto L, Martelletti P. A two years open-label prospective study of OnabotulinumtoxinA 195 U in medication overuse headache: a real-world experience. *J Headache Pain*. 2015;17:1. doi:10.1186/s10194-016-0591-3
310. D'Amico D, Grazzi L, Bussone G, et al. Are depressive symptomatology, self-efficacy, and perceived social support related to disability and quality of life in patients with chronic migraine associated to medication overuse? Data from a cross-sectional study. *Headache*. 2015;55(5):636-645. doi:10.1111/head.12534
311. Cerritelli F, Ginevri L, Messi G, et al. Clinical effectiveness of osteopathic treatment in chronic migraine: 3-Armed randomized controlled trial. *Complement Ther Med*. 2015;23(2):149-156. doi:10.1016/j.ctim.2015.01.011
312. Gori S, Lucchesi C, Baldacci F, Bonuccelli U. Preferential occurrence of attacks during night sleep and/or upon awakening negatively affects migraine clinical presentation. *Funct Neurol*. 2015;30(2):119-123. doi:10.11138/fneur/2015.30.2.119
313. Raggi A, Schiavolin S, Leonardi M, et al. Approaches to treatments of chronic migraine associated with medication overuse: a comparison between different intensity regimens. *Neurol Sci*. 2015;36 Suppl 1:5-8. doi:10.1007/s10072-015-2134-0
314. Condello C, Piano V, Dadam D, Pinessi L, Lantéri-Minet M. Pain beliefs and perceptions inventory: a cross-sectional study in chronic and episodic migraine. *Headache*. 2015;55(1):136-148. doi:10.1111/head.12503
315. de Roos NM, Giezenaar CG, Rovers JM, Witteman BJ, Smits MG, van Hemert S. The effects of the multispecies probiotic mixture Ecologic®Barrier on migraine: results of an open-label pilot study. *Benef Microbes*. 2015;6(5):641-646. doi:10.3920/BM2015.0003
316. Yang Y, Huang X, Fan Y, Wang Y, Ma K. Efficacy of Pulsed Radiofrequency on Cervical 2-3 Posterior Medial Branches in Treating Chronic Migraine: A Randomized, Controlled, and Double-Blind Trial. *Evid Based Complement Alternat Med*. 2015;2015:690856. doi:10.1155/2015/690856
317. Domingues RB, Duarte H, Rocha NP, Teixeira AL. Neurotrophic factors in tension-type headache. *Arq Neuropsiquiatr*. 2015;73(5):420-424. doi:10.1590/0004-282X20150000
318. Boudreau GP, Grosberg BM, McAllister PJ, Lipton RB, Buse DC. Prophylactic onabotulinumtoxinA in patients with chronic migraine and comorbid depression: An open-label, multicenter, pilot study of efficacy, safety and effect on headache-related disability, depression, and anxiety. *Int J Gen Med*. 2015;8:79-86. Published 2015 Feb 18. doi:10.2147/IJGM.S70456

**TTH**

1. Fuensalida-Novo S, Parás-Bravo P, Jiménez-Antona C, et al. Gender differences in clinical and psychological variables associated with the burden of headache in tension-type headache. *Women Health*. 2020;60(6):652-663. doi:10.1080/03630242.2019.1696440
2. Benito-González E, Palacios-Ceña M, Fernández-Muñoz JJ, et al. Variables associated with sleep quality in chronic tension-type headache: A cross-sectional and longitudinal design. *PLoS One*. 2018;13(5):e0197381. Published 2018 May 17. doi:10.1371/journal.pone.0197381
3. Katsarava Z, Mania M, Lampl C, Herberhold J, Steiner TJ. Poor medical care for people with migraine in Europe - evidence from the Eurolight study. *J Headache Pain*. 2018;19(1):10. Published 2018 Feb 1. doi:10.1186/s10194-018-0839-1
4. Cigarán-Méndez M, Fernández-Muñoz JJ, Navarro-Pardo E, et al. Gender differences in variables associated with sleep quality in chronic tension type headache. *Women Health*. 2018;58(9):1037-1049. doi:10.1080/03630242.2017.1372845
5. Palacios-Ceña M, Barbero M, Falla D, Ghirlanda F, Arend-Nielsen L, Fernández-de-Las-Peñas C. Pain Extent Is Associated with the Emotional and Physical Burdens of Chronic Tension-Type Headache, but Not with Depression or Anxiety. *Pain Med*. 2017;18(10):2033-2039. doi:10.1093/pm/pnx047
6. Palacios-Ceña M, Castaldo M, Wang K, et al. Relationship of active trigger points with related disability and anxiety in people with tension-type headache. *Medicine (Baltimore)*. 2017;96(13):e6548. doi:10.1097/MD.0000000000006548
7. Kanji G, Weatherall M, Peter R, Purdie G, Page R. Efficacy of regular sauna bathing for chronic tension-type headache: a randomized controlled study. *J Altern Complement Med*. 2015;21(2):103-109. doi:10.1089/acm.2013.0466
8. Bernard XW et al. Path Analysis Models Integrating Psychological, Psycho-physical and Clinical Variables in Individuals with Tension-Type Headache. *The Journal of Pain*. 2023 24 (3) 426-436 doi.org/10.1016/j.jpain.2022.10.003.
9. Cabanillas-Barea S, Ceballos-Laita L, Pérez-Guillén S, et al. The Addition of Diacutaneous Fibrolysis to a Pharmacological Intervention in Patients with Tension-Type Headache: A Randomized Controlled Trial. *J Clin Med*. 2022;11(22):6716. Published 2022 Nov 13. doi:10.3390/jcm11226716
10. Fernández-de-Las-Peñas C, Palacios-Ceña M, Valera-Calero JA, et al. Understanding the interaction between clinical, emotional and psychophysical outcomes underlying tension-type headache: a network analysis approach. *J Neurol*. 2022;269(8):4525-4534. doi:10.1007/s00415-022-11039-5
11. González de la Flor Á, García Pérez de Sevilla G, Domíngez Balmaseda D, Martín Vera D, Montero Martínez M, Del Blanco Muñiz JÁ. Relationship between Self-Efficacy and Headache Impact, Anxiety, and Physical Activity Levels in Patients with Chronic Tension-Type Headache: An Observational Study. *Behav Neurol*. 2022;2022:8387249. Published 2022 Sep 6. doi:10.1155/2022/8387249
12. Corum M, Aydin T, Medin Ceylan C, Kesiktas FN. The comparative effects of spinal manipulation, myofascial release and exercise in tension-type headache patients with neck pain: A randomized controlled trial. *Complement Ther Clin Pract*. 2021;43:101319. doi:10.1016/j.ctcp.2021.101319
13. Kim KM, Kim J, Cho SJ, et al. Excessive Daytime Sleepiness in Tension-Type Headache: A Population Study. *Front Neurol*. 2019;10:1282. Published 2019 Dec 3. doi:10.3389/fneur.2019.01282
14. Kim J, Cho SJ, Kim WJ, Yang KI, Yun CH, Chu MK. Insomnia in tension-type headache: a population-based study. *J Headache Pain*. 2017;18(1):95. Published 2017 Sep 12. doi:10.1186/s10194-017-0805-3
15. Flynn N. Effect of an Online Hypnosis Intervention in Reducing Migraine Symptoms: A Randomized Controlled Trial. *Int J Clin Exp Hypn*. 2019;67(3):313-335. doi:10.1080/00207144.2019.1612674
16. Fernández-de-Las-Peñas C, Benito-González E, Palacios-Ceña M, Wang K, Castaldo M, Arendt-Nielsen L. Identification of subgroups of patients with tension type headache with higher widespread pressure pain hyperalgesia. *J Headache Pain*. 2017;18(1):43. doi:10.1186/s10194-017-0751-0
17. Domingues RB, Duarte H, Rocha NP, Teixeira AL. Neurotrophic factors in tension-type headache. *Arq Neuropsiquiatr*. 2015;73(5):420-424. doi:10.1590/0004-282X20150000

**TACs**

1. Liaw YC, Wang YF, Chen WT, et al. Sex-related differences in cluster headache: A hospital-based study in Taiwan. *Cephalalgia*. 2022;42(14):1532-1542. doi:10.1177/03331024221120054
2. Kamm K, Straube A, Ruscheweyh R. Cluster Headache Impact Questionnaire (CHIQ) - a short measure of cluster headache related disability. *J Headache Pain*. 2022;23(1):37. Published 2022 Mar 18. doi:10.1186/s10194-022-01406-y
3. Merli E, Asioli GM, Favoni V, et al. Great occipital nerve long-acting steroid injections in cluster headache therapy: an observational prospective study. *J Neurol*. 2022;269(4):2193-2199. doi:10.1007/s00415-021-10884-0
4. Fontaine D, Blond S, Lucas C, et al. Occipital nerve stimulation improves the quality of life in medically-intractable chronic cluster headache: Results of an observational prospective study. *Cephalalgia*. 2017;37(12):1173-1179. doi:10.1177/0333102416673206
5. Gil-Martínez A, Navarro-Fernández G, Mangas-Guijarro MÁ, Díaz-de-Terán J. Hyperalgesia and Central Sensitization Signs in Patients with Cluster Headache: A Cross-Sectional Study. *Pain Med*. 2019;20(12):2562-2570. doi:10.1093/pm/pnz070
6. Aschehoug I, Bratbak DF, Tronvik EA. Long-Term Outcome of Patients With Intractable Chronic Cluster Headache Treated With Injection of Onabotulinum Toxin A Toward the Sphenopalatine Ganglion - An Observational Study. *Headache*. 2018;58(10):1519-1529. doi:10.1111/head.13398
7. Barloese MC, Jürgens TP, May A, et al. Cluster headache attack remission with sphenopalatine ganglion stimulation: experiences in chronic cluster headache patients through 24 months. *J Headache Pain*. 2016;17(1):67. doi:10.1186/s10194-016-0658-1

**Primary Headache**

1. Sobe H, Richter M, Berner R, et al. Functional improvement in children and adolescents with primary headache after an interdisciplinary multimodal therapy program: the DreKiP study. *J Headache Pain*. 2022;23(1):109. Published 2022 Aug 25. doi:10.1186/s10194-022-01481-1
2. Ruscheweyh R, Klonowski T, Goßrau G, et al. The headache registry of the German Migraine and Headache Society (DMKG): baseline data of the first 1,351 patients [published correction appears in J Headache Pain. 2022 Jul 15;23(1):82]. *J Headache Pain*. 2022;23(1):74. Published 2022 Jul 1. doi:10.1186/s10194-022-01447-3
3. Ishii R, Schwedt TJ, Trivedi M, et al. Mild traumatic brain injury affects the features of migraine. *J Headache Pain*. 2021;22(1):80. Published 2021 Jul 22. doi:10.1186/s10194-021-01291-x
4. Park HK, Chu MK, Oh SY, et al. Interim analysis of the Registry for Load and Management of Medication Overuse Headache (RELEASE): A multicenter, comprehensive medication overuse headache registry. *Cephalalgia*. 2022;42(6):455-465. doi:10.1177/03331024211057184
5. Delussi M, Laporta A, Fraccalvieri I, de Tommaso M. Osmophobia in primary headache patients: associated symptoms and response to preventive treatments. *J Headache Pain*. 2021;22(1):109. Published 2021 Sep 18. doi:10.1186/s10194-021-01327-2

**Migraine + TTH**

1. Moyes C, Belaghi R, Webster RJ, Whitley N, Pohl D. Cognitive Behavioral Therapy for Children With Headaches: Will an App Do the Trick? *J Child Neurol*. 2023;38(3-4):169-177. doi:10.1177/08830738231170067
2. Tchivileva IE, Ohrbach R, Fillingim RB, et al. Clinical, psychological, and sensory characteristics associated with headache attributed to temporomandibular disorder in people with chronic myogenous temporomandibular disorder and primary headaches. *J Headache Pain*. 2021;22(1):42. Published 2021 May 22. doi:10.1186/s10194-021-01255-1
3. Suzuki K, Suzuki S, Haruyama Y, Kobashi G, Shiina T, Hirata K. Restless legs syndrome is associated with headache-related disabilities in patients with migraine: a prospective 7-year follow-up study. *Eur J Neurol*. 2019;26(2):238-245. doi:10.1111/ene.13796
4. Bottiroli S, Allena M, Sances G, et al. Psychological, clinical, and therapeutic predictors of the outcome of detoxification in a large clinical population of medication-overuse headache: A six-month follow-up of the COMOESTAS Project. *Cephalalgia*. 2019;39(1):135-147. doi:10.1177/0333102418783317
5. Smitherman TA, Davis RE, Walters AB, Young J, Houle TT. Anxiety sensitivity and headache: diagnostic differences, impact, and relations with perceived headache triggers. *Cephalalgia*. 2015;35(8):710-721. doi:10.1177/0333102414557840
6. Qin T, Chen C. Cognitive Dysfunction in Migraineurs. *Medicina (Kaunas)*. 2022;58(7):870. Published 2022 Jun 29. doi:10.3390/medicina58070870
7. Olivier S, Antonioni A, Mezzetta B, et al. A trial to prove the efficacy of acupuncture as a therapeutic support in pharmacological prophylaxis for migraine and tension-type headache. Pilot study. *Confinia Cephalalgica*. 2022
8. Shimizu T, Sakai F, Miyake H, et al. Disability, quality of life, productivity impairment and employer costs of migraine in the workplace. *J Headache Pain*. 2021;22(1):29. Published 2021 Apr 21. doi:10.1186/s10194-021-01243-5
9. Gil-Gouveia R, Marques IB, Parreira EP, Martins IP, Oliveira AG. Headache Gauge: a real-life calendar-based tool for headache monitoring. *Neurol Sci*. 2021;42(10):4163-4174. doi:10.1007/s10072-021-05080-x
10. Vasiliou VS, Karademas EC, Christou Y, Papacostas S, Karekla M. Acceptance and Commitment Therapy for Primary Headache Sufferers: A Randomized Controlled Trial of Efficacy. *J Pain*. 2021;22(2):143-160. doi:10.1016/j.jpain.2020.06.006
11. Cho S, Lee MJ, Park HR, Kim S, Joo EY, Chung CS. Effect of Sleep Quality on Headache-Related Impact in Primary Headache Disorders. *J Clin Neurol*. 2020;16(2):237-244. doi:10.3988/jcn.2020.16.2.237
12. Albanês Oliveira Bernardo A, Lys Medeiros F, Sampaio Rocha-Filho PA. Osmophobia and Odor-Triggered Headaches in Children and Adolescents: Prevalence, Associated Factors, and Importance in the Diagnosis of Migraine. *Headache*. 2020;60(5):954-966. doi:10.1111/head.13806
13. Viana M, De Icco R, Allena M, et al. Clinical Subtypes of Medication Overuse Headache - Findings From a Large Cohort. *Headache*. 2019;59(9):1481-1491. doi:10.1111/head.13641
14. Fernández-de-Las-Peñas C, Ambite-Quesada S, Palacios-Ceña M, et al. Catechol-O-Methyltransferase (COMT) rs4680 Val158Met Polymorphism is Associated with Widespread Pressure Pain Sensitivity and Depression in Women With Chronic, but not Episodic, Tension-Type Headache. *Clin J Pain*. 2019;35(4):345-352. doi:10.1097/AJP.0000000000000684
15. Ishiyama S, Shibata Y, et al. A. Clinical Effect of C2 Peripheral Nerve Field Stimulation Using Electroacupuncture for Primary Headache. *Neuromodulation* 2018 793 – 796 doi: 10.1111/ner.12772
16. Bougea A, Spantideas N, Lyras V, Avramidis T, Thomaidis T. Melatonin 4 mg as prophylactic therapy for primary headaches: a pilot study. *Funct Neurol*. 2016;31(1):33-37. doi:10.11138/fneur/2016.31.1.033
17. Kemper KJ, Heyer G, Pakalnis A, Binkley PF. What Factors Contribute to Headache-Related Disability in Teens? *Pediatr Neurol*. 2016; 56:48-54. doi: 10.1016/j.pediatrneurol.2015.10.024

**Migraine + TTH + TACs**

1. Shaygan M, Rahmanian S, et al. Predictive Factors for Disability in Patients with Acute and Chronic Headache. *Shiraz E-Medical Journal*. 2019 20. 10.5812/semj.87499.

**Migraine + TACs**

1. Miller S, Watkins L, Matharu M. Long-term outcomes of occipital nerve stimulation for chronic migraine: a cohort of 53 patients. *J Headache Pain*. 2016;17(1):68. doi:10.1186/s10194-016-0659-0
